# Genome-wide identification and expression profile analysis of metal tolerance protein gene family in *Eucalyptus grandis* under metal stresses

**DOI:** 10.1186/s12870-023-04240-9

**Published:** 2023-05-06

**Authors:** Zahra Shirazi, Fatemeh Khakdan, Fariba Rafiei, Mahdi Yahyazadeh Balalami, Mojtaba Ranjbar

**Affiliations:** 1 Department of Biotechnology Research, Research Institute of Forests and Rangelands, Agricultural Research, Education and Extension Organization (AREEO), National Botanical Garden, Tehran Karaj Freeway, P.O. Box 13185-116, Tehran, Iran; 2 Farzanegan Campus, Semnan University, Semnan, Iran; 3 Department of Agronomy, Purdue University, West Lafayette, IN 47907 USA; 4 Department of Medicinal Plant Research, Research Institute of Forests and Rangelands, Agricultural Research, Education and Extension Organization (AREEO), National Botanical Garden, Tehran Karaj Freeway, P.O. Box 13185-116, Tehran, Iran; 5 Microbial Biotechnology Department, College of Biotechnology, University of Special Modern Technologies, Amol, Iran

**Keywords:** Genome-wide identification, *Eucalyptus grandis*, Heavy metals, Metal tolerance protein (MTP), Expression profile

## Abstract

Metal tolerance proteins (MTPs) as Me^2+^/H^+^(K^+^) antiporters participate in the transport of divalent cations, leading to heavy metal stress resistance and mineral utilization in plants. In the present study, to obtain better knowledge of the biological functions of the MTPs family, 20 potential *EgMTPs* genes were identified in *Eucalyptus grandis* and classified into seven groups belonging to three cation diffusion facilitator groups (Mn-CDFs, Zn/Fe-CDFs, and Zn-CDFs) and seven groups. *EgMTP*-encoded amino acids ranged from 315 to 884, and most of them contained 4–6 recognized transmembrane domains and were clearly prognosticated to localize into the cell vacuole. Almost all *EgMTP* genes experienced gene duplication events, in which some might be uniformly distributed in the genome. The numbers of cation efflux and the zinc transporter dimerization domain were highest in EgMTP proteins. The promoter regions of *EgMTP* genes have different cis-regulatory elements, indicating that the transcription rate of *EgMTP* genes can be a controlled response to different stimuli in multiple pathways. Our findings provide accurate perception on the role of the predicted miRNAs and the presence of SSR marker in the Eucalyptus genome and clarify their functions in metal tolerance regulation and marker-assisted selection, respectively. Gene expression profiling based on previous RNA-seq data indicates a probable function for *EgMTP* genes during development and responses to biotic stress. Additionally, the upregulation of *EgMTP6*, *EgMTP5*, and *EgMTP11.1* to excess Cd^2+^ and Cu^2+^ exposure might be responsible for metal translocation from roots to leaves.

**Supplementary Information:**

The online version contains supplementary material available at 10.1186/s12870-023-04240-9.

## Background

Some specified metal ions, including Zn^2+^, Mn^2+^, Fe^2+^, Co^2+^, Cd^2+^, and Cu^2+^, are needed in plant cells for numerous low-level cellular, physiological, and metabolic reactions [1]. They greatly affect many enzymes and regulatory proteins functioning in plant cells to carry out many particular physiological and biochemical processes and increase agricultural productivity, and a deficiency in these ions negatively influences the plant growth and development [2, 3].

Moreover, a deficient concentration of non-essential metal ions, including Ag^+^, Cd^+2^, Cr^+2^, Pb^+2^, Al^+2^, and Hg^+2^, have no known functions in plants and can also cause plant cell toxicity at very low concentrations [4]. In addition to the negative subsequence in plant cell toxicity, some of these non-essential metals were considered to be a universal environmental matter for the human health when stored in crop plants grown on polluted soil used for food [5, 6]. They can enter the food chain through crops and accumulate in the human body through biomagnification, thus posing a great threat to human health, such as gastrointestinal and kidney dysfunction, nervous system disorders, skin lesions, vascular damage, immune system dysfunction, birth defects, and cancer [7].

Diverse remediation approaches with mechanical or physio-chemical strategies are utilized to reclaim heavy metal-contaminated soil with inefficient mechanical, physical, and chemical strategies, expensive equipment, and poisonous chemicals which can deteriorate the soil ecosystem and introduce of secondary pollutions [8]. Therefore, many researchers are trying to develop an expanding pattern to employ the plant to remove elemental pollutants known as "phytoremediation". In this field, trees are known to be bio-monitors, especially in urban areas in which the concentration of heavy metals is monitored by accumulating them in various organelles present in roots, fruits, barks and leaves. Trees are able to accumulate heavy metals and modulate their bioavailability in soil through the extension of their root system [9]. This technique gives various advantages like (i) being economically feasible, (ii) being eco-friendly, (iii) being applicable, (iv) preventing erosion and metal leaching, and (v) improving soil fertility. Over the previous decades, many studies have been performed to discover the molecular mechanisms involved in heavy metal tolerance and develop approaches to increase phytoremediation efficiency [10].

Plants have numerous physiological and molecular mechanisms as a part of the response-specific metal toxicity to precisely regulate cellular concentrations of metal ions, i.e. metal efflux and uptake, chelation, storage, trafficking, and detoxification/sequestration [11]. In plant cells, vacuole is an important detoxication, cessation, and storage site for overabundance metal ions [12], and its membrane contains different types of protein transporter families with specific transporting ions. In both prokaryotic and eukaryotic cells, cation diffusion facilitators (CDFs) gene family encode mainly Me^2+^/H^+^ integral membrane ion transport proteins, which act as divalent cation transporters in the transportation of some ions such as cadmium, iron, zinc, manganese, cobalt, and nickel, from the cytosol into organelles [13]. The CDFs family transporters have been divided into three clusters: 1) Zn/Fe-CDF, Mn-CDF, and Zn-CDF. Most CDF transport proteins contain modified signature domains; 4 to 6 transmembrane domains (TMDs) and ion transport domain at the C-terminus [14]. The first group includes phyla specific members such as the Zrc1-like, DmeF-like, ZitB-like, the ZnT1-like, and ZnT6-like clusters, which is a broad specificity transporter with a preference for Co^2+^ and Zn^2+^, and the EcZitB protein from *E. coli*, which mainly transports Zn^2+^ and Cd^2+^. Two groups contain Fe/Zn-CDF transporters (MMT-like, FieF-like, and WmFieF-like clusters) and the third encloses only the Mn-CDF transporter (MTP8-like sequences) which shares many conserved residues, suggesting that they are derived from a common ancestor [15]. These transmembrane domains are commonly interlinked by interconnection loops as extra- and intracellular patterns, in which their cytosolic parts generally contain a domain that is rich in histidine, which is known as a metal binding domain [15].

CDF transporters in plant cells are commonly considered metal tolerance proteins (MTPs), which can be clustered in seven distinct phylogenetic classes, according to the exclusivities in transported special metals: Zn-CDFs in groups 1–4, 5, and 12 function as MTP1-MTP4, MTP5, and MTP12, groups 6 and 7 function as Fe/Zn-CDFs containing MTP6 and MTP7, and Mn-CDFs are placed in group 8 (MTP8) and 9 (MTP9-MTP11) [16]. MTP proteins have also been previously identified in *Oryza sativa* [17], *Triticum aestivum* [17], *Populus trichocarpa* [18], *Medicago truncatula*, *Arachis hypogaea* [19], *Nicotiana tomentosiformis* [20], and *Vitis vinifera* [21]. Previous reports have shown that Zn-CDFs play a fundamental role in transporting both Zn and other metallic cations. For instance, the MTP1 and MTP3 proteins from *Arabidopsis thaliana* (AtMTP1 and AtMTP3) were considered to be Co^2+^ and/or Zn ions transporters by their excess transferring of ions into the vacuole [22].

Regarding rice OsMTP1, it has been reported that MTP1 is presumably involved in the translocation of non- and essential divalent metals like Cd^+2^, Co^+2^, Zn^+2^, and Fe^+2^, resulting in the homeostasis of these metals in plant cells [23]. Moreover, AtMTP3 and AtMTP12 have been established to be localized in the vacuole as a heterodimer functional complex involved in the translocation of Zn from the cytosol to the Golgi bodies [24] as AtMTP8, AtMTP11, OsMTPs (8.1, 8.2, 9, 11, 11.1), ShMTP8, CsMTP8, HvMTP8.1, CiMTP8, and TaMTP8. The Mn transporter members play a pivotal role in the transportation of Mn into the vacuole or Golgi bodies and maintain the Mn homeostasis in different plant species, resulting in the protection of plant cells from excessive endoplasmic vesicles [25, 26]. In *Vitis viniera*, the VvMTP1- VvMTP12 of the cell vacuole, participate in the transportation of metal ions producing environmental stress responses, particularly hyperosmotic stress [21]. However, Delhaize et al. [25] reported that the overexpression of *ShMTP* gene from *Stylosanthes hamata* resulted in Mn tolerance in Arabidopsis. Additionally, the members of the heavy metals tolerance genes family were identified in *P. trichocarpa,* and their influence on reaction to heavy metals was screened [18].

*Eucalyptus grandis* (2n = 4x = 40), one of the top 500 species of Eucalyptus widely grown on saline and alkaline lands across the world, has received great attention to produce high-valued medicinal oil by aromatic leaves and timber [27]. Previous studies have demonstrated that *Eucalyptus grandis* seedlings have a high capacity to accumulate heavy metal pollution in their root, as an adaptation system with hardly detrimental conditions, which would enable them to survive and bioaccumulate in response to heavy metals [8]. Its complete genome has been sequenced, facilitating the classification and comparative genomics and providing a chance to display candidate genes. Additionally, the whole-genome sequences of *Eucalyptus grandis* provided an opportunity to analyze the *EgMTP* gene family at the genome-wide level.

Irrespective of several researches on evaluating the variation of metal tolerance and accumulation in the well-known species of *Eucalyptus*, according to our knowledge, there is a research gap in identifying the *EgMTP* genes family in *Eucalyptus grandis* and discovering their structure and evolutionary relationships of these sequences. To facilitate the perception of the potential roles of Eucalyptus MTPs, the expression profiling of known genes was evaluated in response to two important divalent metals (Cd^2+^ and Cu^2+^). This research aims to analyze the evolution of the *MTP* gene family in *Eucalyptus grandis* and understand further functional characterization of the MTP gene family in both plant compartments (roots and leaves) and response to heavy metal stress in a plant cell, which will provide a novel research condition to understand the molecular mechanism of metal transport and homeostasis and eventually will help to identify the tolerant- heavy metal cultivars or species in future works.

## Results

### Identification of *MTP* genes in *E. grandis*

Homology search for the identification of the *MTP* gene family in the genome of *E. grandis* was performed based on the *MTP* gene sequences in *A. thaliana* [28] and *O. sativa* [17] using BLASTP. Subsequently, forty-seven MTP candidate proteins with cation efflux domain (PF01545) verified by HMMER-EMBL-EBI database [29] were identified in the *E. grandis* genome. Finally, twenty MTP proteins of *Eucalyptus,* based on high similarities with the *Arabidopsis* MTP families, were designated for subsequent analysis. The candidate genes were nominated with a particular name, i.e. *EgMTP1.4*, *EgMTP1.6*, *EgMTP1.5*, *EgMTP1.2*, *EgMTP1.1*, *EgMTP1.3*, *EgMTP11.2*, *EgMTP12*, *EgMTP2*, *EgMTP5*, *EgMTP1.7*, *EgMTP9.2*, *EgMTP7*, *EgMTP9.1*, *EgMTP9.3*, *EgMTP8.1*, *EgMTP4*, *EgMTP6*, *EgMTP8.2*, and* EgMTP11.1*. The features of the EgMTPs protein sequences were investigated in details (Table 1). The length of the encoded amino acid ranged from 315 (*EgMTP9.3*) to 884 (*EgMTP12*), and the molecular weight of EgMTP proteins was from 36.07 kDa (*EgMTP9.*3) to 97.32 kDa (*EgMTP12*). Most of the EgMTPs showed a low isoelectric point from 5.03 (*EgMTP11.1*) to 8.62 (*EgMTP9.3*), 17 *EgMTP* members with low isoelectric point (pI < 7) and 3 *EgMTP* members with a relatively high isoelectric point (pI > 7). The results of predicted TMD numbers with cytosolic N and C termini exhibited a variable range, in which most of the EgMTP protein sequences had 4–6 TMDs (Table 1). As shown in Table 1, all *EgMTP* proteins were anticipated to be localized into the vacuole membrane.

**Table 1:** Identification and characteristics of MTP (metal tolerance protein) in *Eucalyptus grandis*

Gene	Accession number	Peptide length	PI	MW (kDa)	TMD NO. N to C	Subcellular localization
*EgMTP1.4*	Eucgr.E01088	415	5.75	45.85	6/ in to in	Vacuole
*EgMTP1.6*	Eucgr.E01090	421	5.86	46.52	6/ in to in	Vacuole
*EgMTP1.5*	Eucgr.E01082	415	5.77	45.97	6/ in to in	Vacuole
*EgMTP1.2*	Eucgr.E01089	395	5.98	44.10	6/ in to in	Vacuole
*EgMTP1.1*	Eucgr.E01087	405	5.93	44.93	6/ in to in	Vacuole
*EgMTP1.3*	Eucgr.E01084	415	5.82	45.97	6/ in to in	Vacuole
*EgMTP11.2*	Eucgr.J01168	324	5.37	36.68	4/ out to out	Vacuole
*EgMTP12*	Eucgr.J01747	884	6.90	97.32	12/ in to in	Vacuole
*EgMTP2*	Eucgr.C02043	335	5.89	37.53	4/ out to out	Vacuole
*EgMTP5*	Eucgr.A02454	392	8.23	43	6/ in to in	Vacuole
*EgMTP1.7*	Eucgr.D01642	440	5.99	48.58	6/ in to in	Vacuole
*EgMTP9.2*	Eucgr.F04469	389	5.94	44.86	6/ in to in	Vacuole
*EgMTP7*	Eucgr.F04020	473	7.34	51.89	4/ in to in	Vacuole
*EgMTP9.1*	Eucgr.F04468	402	6.75	46.5	6/ in to in	Vacuole
*EgMTP9.3*	Eucgr.F04467	315	8.62	36.07	5/ out to out	Vacuole
*EgMTP8.1*	Eucgr.B03634	401	5.06	44.99	5/ in to out	Vacuole
*EgMTP4*	Eucgr.B02300	385	5.67	42.59	6/ in to in	Vacuole
*EgMTP6*	Eucgr.K03452	507	6.48	55.39	4 in to in	Vacuole
*EgMTP8.2*	Eucgr.K01853	421	5.56	47.02	4/ in to in	Vacuole
*EgMTP11.1*	Eucgr.G02893	401	5.03	45.04	4/ out to out	Vacuole

Gene (gene name based on phylogenetic distribution and sequence similarity to Arabidopsis MTPs using the MatGAT software; MW (predicted molecular weight based on kilo Daltons); pI (predicted isoelectric point); predicted TMDs (no. of the transmembrane domains); in (cytoplasmic) or out (extracellular) predicted from N to C terminus

### Phylogenetic analysis of *MTP* gene families

**Fig. 1:**
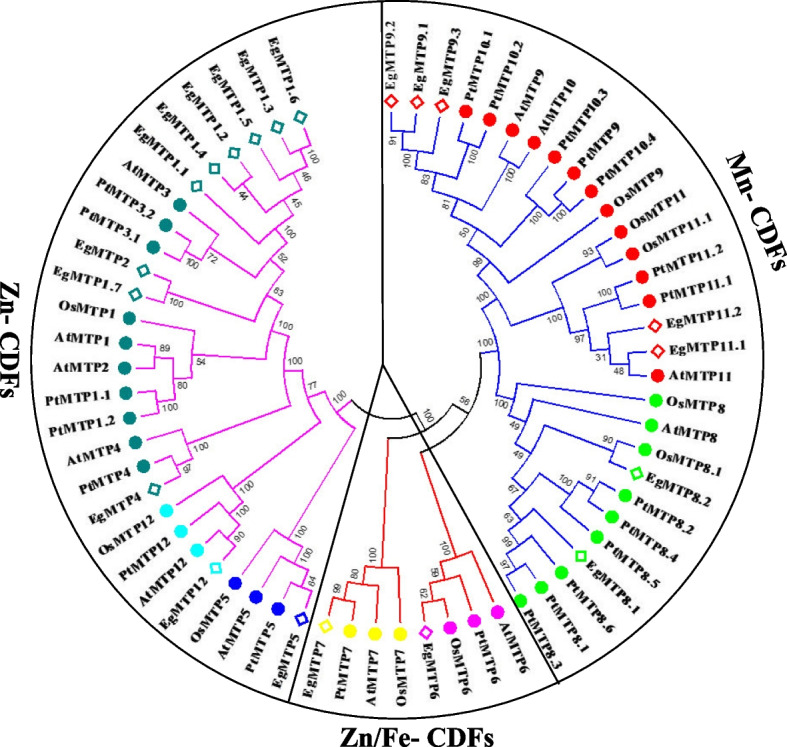
Phylogenetic relationship of MTP proteins family members of *Eucalyptus grandis*, *Populus trichocarpa*, *Arabidopsis thaliana*, and *Oryza sativa*. The MTP protein sequences were aligned by ClustalX 2.0.8, and the phylogenetic trees were constructed based on the analysis of bootstrap with 100 replicates by neighbor-joining method using the MEGA 6.0 program. The identified proteins were classified into three sub-families (Fe/Zn-MTPs, Mn-MTPs, and Zn-MTPs) and seven groups based on the previous reports of phylogenetic relationships. The Zn-MTP group (violet line) contains MTP1 to MTP4, MTP5, and MTP12 groups; the Mn- MTP group (blue line) contains MTP8 and MTP9 to MTP11 groups; and the Zn/Fe- MTP group (red line) contains MTP6 and MTP7 groups

The evolutionary relationship of the MTP proteins of *Eucalyptus grandis*, *Populus trichocarpa*, *Arabidopsis thaliana*, and *Oryza sativa* was surveyed by constructing the phylogenetic tree based on the analysis of bootstrap with 100 replicates by the MEGA 5.2 program and the neighbor-joining method (Fig. 1). All these *MTP* gene families were divided into three major sub-families (Mn-MTPs, Zn/Fe-MTPs, and Zn-MTPs), which contained seven groups, i.e. groups 1–4, 5, 6, 7, 8, 9, and 12 based upon their phylogenetic relationship and previous results reported by Montanini et al. [15]. Of the three sub-families, the highest number of *MTPs* were placed in the *Zn-MTP* sub-families, containing 11 *EgMTPs* (*EgMTP1/1.1/1.2/1.3/1.4/1.5/1.6/1.7, EgMTP2, EgMTP4, EgMTP5*, and *EgMTP12*), and then Mn-MTP sub-families, including *EgMTP8.1/8.2, EgMTP9.1/9.2/9.3, and EgMTP11.1/11*. Finally, two members containing *EgMTP6* and *EgMTP7* were clustered in the *Zn/Fe-MTP* sub-family (Fig. 1).

### Chromosomal mapping, gene duplication, and evaluation analysis

**Fig. 2:**
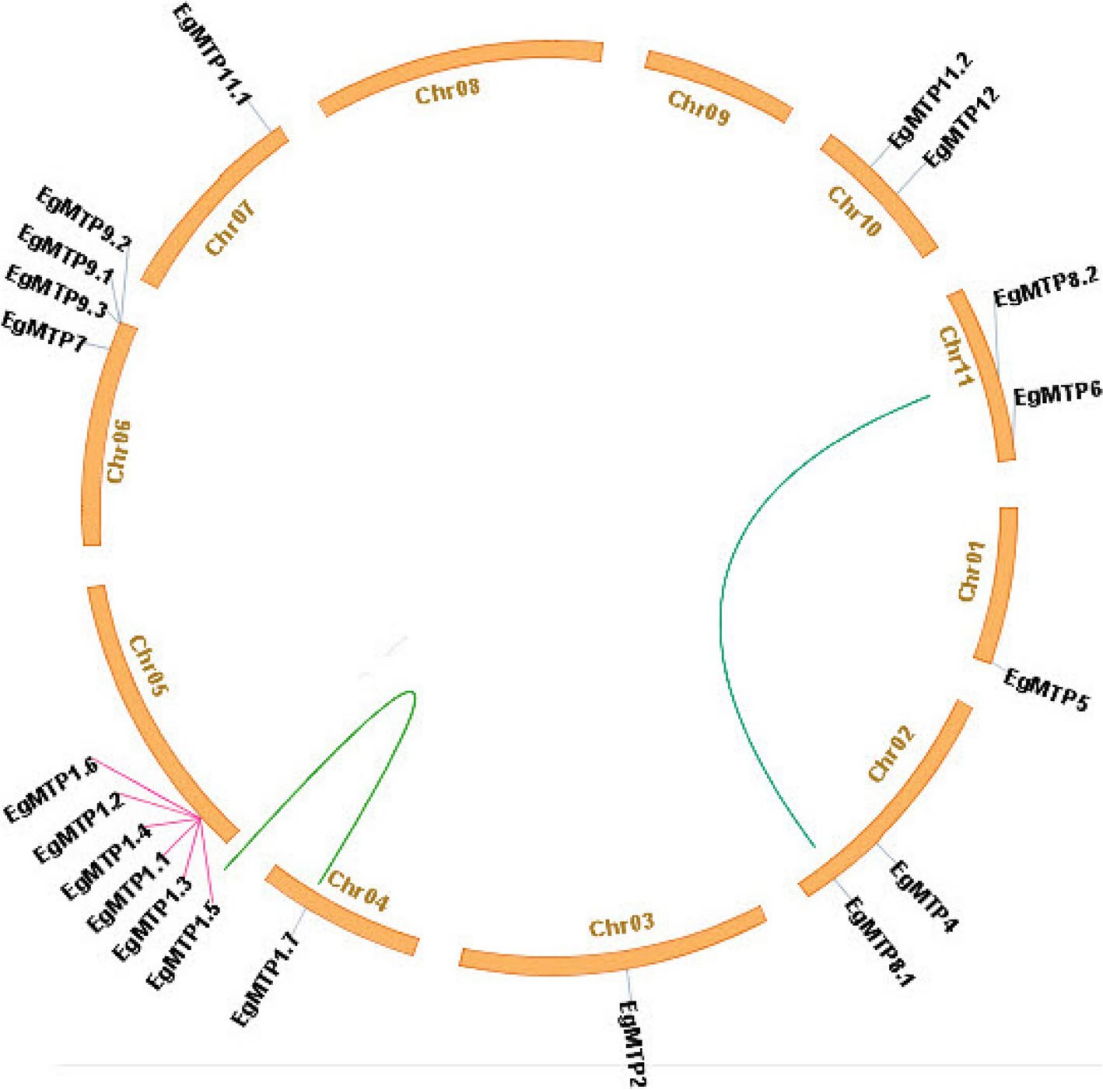
Chromosomal locations and duplications of *EgMTP* genes in the* Eucalyptus grandis* genome. The location of *EgMTP* genes on chromosomes and the duplication relationship between them were revealed using TBtools genetic mapping software. Tandem duplication genes and segmental duplication genes are marked with pink lines and green curves, respectively

The distribution of the *EgMTP* gene family on different chromosomes was identified and performed by TBtools genetic mapping software. The chromosomal location results revealed *EgMTP* genes were observed to be unevenly distributed on nine of the eleven chromosomes (Fig. 2). The maximum number of *EgMTP* genes per chromosome was found at chromosomes 05 and 06, which contained 6 (*EgMTP1.1, 1.2, 1.3, 1.4, 1.5, 1.6*) and 4 (*EgMTP9.1, 9.2, 9.3,* and *EgMTP7*) *EgMTPs*, respectively. Chromosomes 02, 10, and 11 had two genes each, each of the four chromosomes (01, 03, 04, and 07) possessed only one gene, whereas no *EgMTP* was recognized in the chromosomes 08 and 09. Noticeably, all *EgMTP* genes on the same chromosome did not separate at a high distance. Six paralogous pairs of *EgMTP* were located on chromosome 05, which contained* EgMTP1.1* to *EgMTP1.6* in the tandem arrangement with above 80% similarity percentage. As indicated in Table 2, the presence of a few homologies of *MTP* gene family members between chromosomal pairs of *E. grandis was* caused by the segmental duplication event, like what occurred in *EgMTP8.1*/*EgMTP8.2* from the chromosomes 02/11 and *EgMTP1.5*/*EgMTP1.7* from the chromosomes 04/05. Additionally, tandem duplication was detected among almost more *EgMTP* genes, which included *EgMTP1.5/ EgMTP1.3*, *EgMTP1.5/ EgMTP1.1*, *EgMTP1.5/ EgMTP1.4*, *EgMTP1.5/ EgMTP1.2*, *EgMTP1.5/ EgMTP1.6*, *EgMTP1.3/ EgMTP1.1*, *EgMTP1.3/ EgMTP1.4*, *EgMTP1.3/ EgMTP1.2*, *EgMTP1.3/ EgMTP1.6, EgMTP1.1/ EgMTP1.4*, *EgMTP1.1/ EgMTP1.2*, *EgMTP1.1/ EgMTP1.6*, *EgMTP1.4/ EgMTP1.2*, *EgMTP1.4/ EgMTP1.6*, and *EgMTP1.2/ EgMTP1.6.* The ratio of Ka and Ks of all gene duplication pairs was less than 1 (Table 2). The maximum transitional substitution (13.66%**)** in 20 nucleotide sequences covered T/U to C. The mean intra- and inter-group differences among the three sub-families showed the maximum difference between Mn-MTPs and Zn-MTPs and the minimum difference in Mn-MTPs.

**Table 2:** Estimated divergence between paralogous *MTP* gene pairs in *Eucalyptus grandis*

Gene 1	Gene 2	Duplicate Type	Ka	Ks	Ka/Ks	Selection
*EgMTP1.5*	*EgMTP1.7*	Segmental	1.2020	0.1652	0.0131	Purifying
*EgMTP8.1*	*EgMTP8.2*	Segmental	0.1394	2.0094	0.0694	Purifying
*EgMTP1.5*	*EgMTP1.3*	Tandem	0.0747	0.0349	0.0832	Purifying
*EgMTP1.5*	*EgMTP1.1*	Tandem	0.1620	1.9117	0.0847	Purifying
*EgMTP1.5*	*EgMTP1.4*	Tandem	0.0826	0.0245	0.0848	Purifying
*EgMTP1.5*	*EgMTP1.2*	Tandem	0.0749	0.0328	0.0162	Purifying
*EgMTP1.5*	*EgMTP1.6*	Tandem	0.0751	0.0286	0.1150	Purifying
*EgMTP1.3*	*EgMTP1.1*	Tandem	0.0994	0.0348	0.2089	Purifying
*EgMTP1.3*	*EgMTP1.4*	Tandem	0.0751	0.0349	0.2366	Purifying
*EgMTP1.3*	*EgMTP1.2*	Tandem	0.0749	0.0519	0.2865	Purifying
*EgMTP1.3*	*EgMTP1.6*	Tandem	0.0218	0.0491	0.0101	Purifying
*EgMTP1.1*	*EgMTP1.4*	Tandem	0.0218	0.0182	0.3440	Purifying
*EgMTP1.1*	*EgMTP1.2*	Tandem	0.0992	0.0391	0.4180	Purifying
*EgMTP1.1*	*EgMTP1.6*	Tandem	0.1082	0.0285	0.4906	Purifying
*EgMTP1.4*	*EgMTP1.2*	Tandem	0.0908	0.0245	0.5175	Purifying
*EgMTP1.4*	*EgMTP1.6*	Tandem	0.0834	0.0286	0.5178	Purifying
*EgMTP1.2*	*EgMTP1.6*	Tandem	0.0674	0.0	0.5616	Purifying

Ka (non-synonymous substitution rate); Ks (synonymous substitution rate); Ka / Ks = 1 (natural selection); Ka / Ks > 1 (positive selection); Ka / Ks < 1 (purifying selection)

### Gene structures and conserved motif

**Fig. 3:**
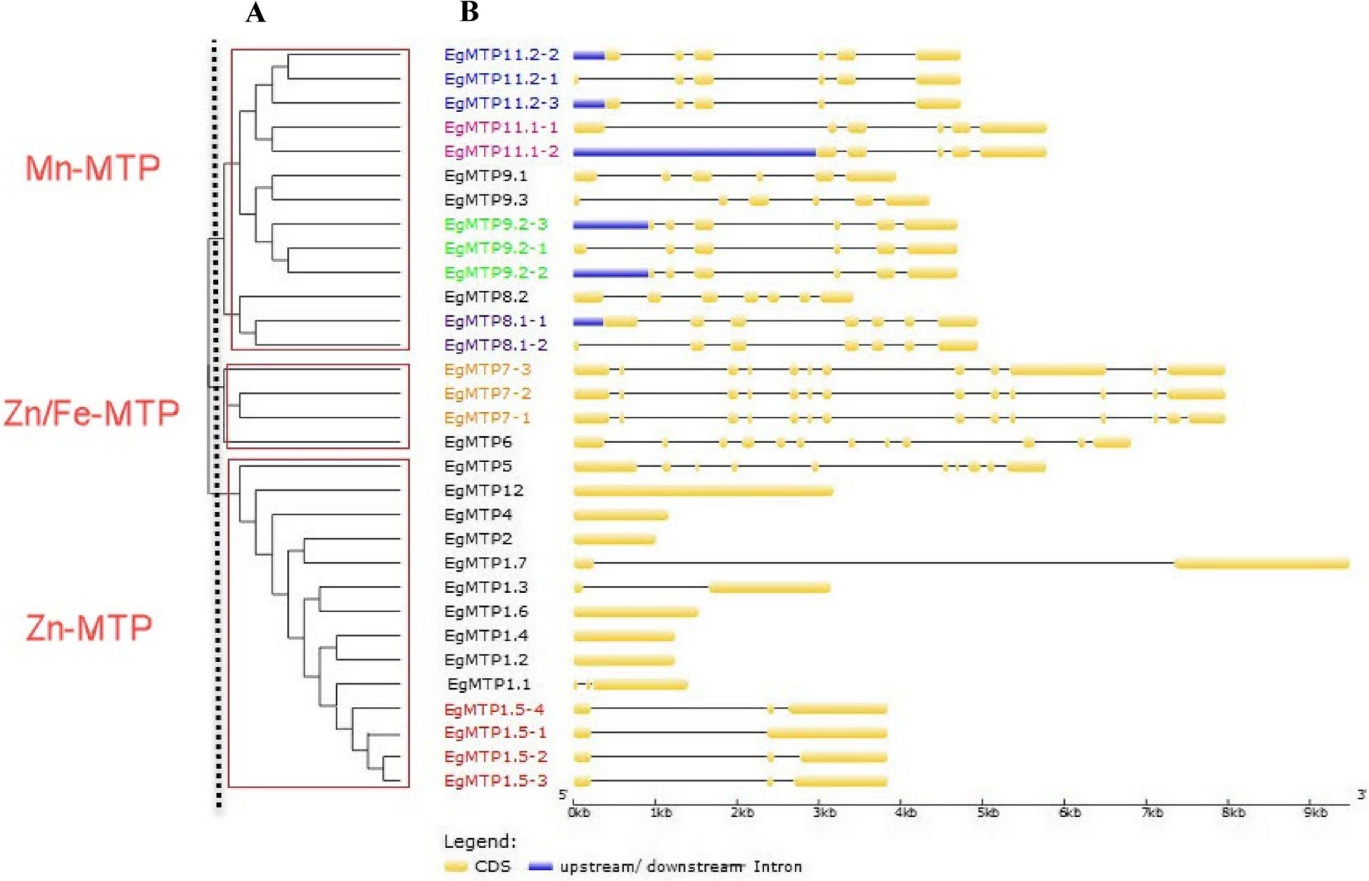
Phylogenetic relationship and gene structures (exon–intron organization) of *EgMTP* genes. A) the neighbor joining phylogenetic tree was constructed using MEGA 6.0 with 1000 times replication. B) the exon–intron structures of EgMTP proteins, where coding DNA sequences are shown with yellow boxes. Also, thick blue lines at either terminal of the genes indicate untranslated regions (UTRs) and thin lines show introns. The same colors in the names of the genes indicate alternative splicing forms

To understand the evolutionary relationship of the *EgMTP* family, the organizations of exon–intron of *EgMTP* genes were investigated (Fig. 3 A and B). Figure 3 A and B show that the *EgMTP* gene family contains 14 exons. The *EgMTP11.2*, *EgMTP7.2*, and *EgMTP9.2* contain three alternative splicing isoforms, while *EgMTP8.1* and *EgMTP11.1* include two and *EgMTP1.5* four alternative splicing isoforms, respectively. The maximum association was observed within the sub-family, while exon numbers displayed the maximum structural variety among the *EgMTP* sub-families. The highest uniformity was recorded in the Mn-MTP sub-family with 5 to 7 exons, while the Zn-MTP sub-family with 1 to 10 exons contained the lowest number (Fig. 3 A and B).

**Fig. 4:**
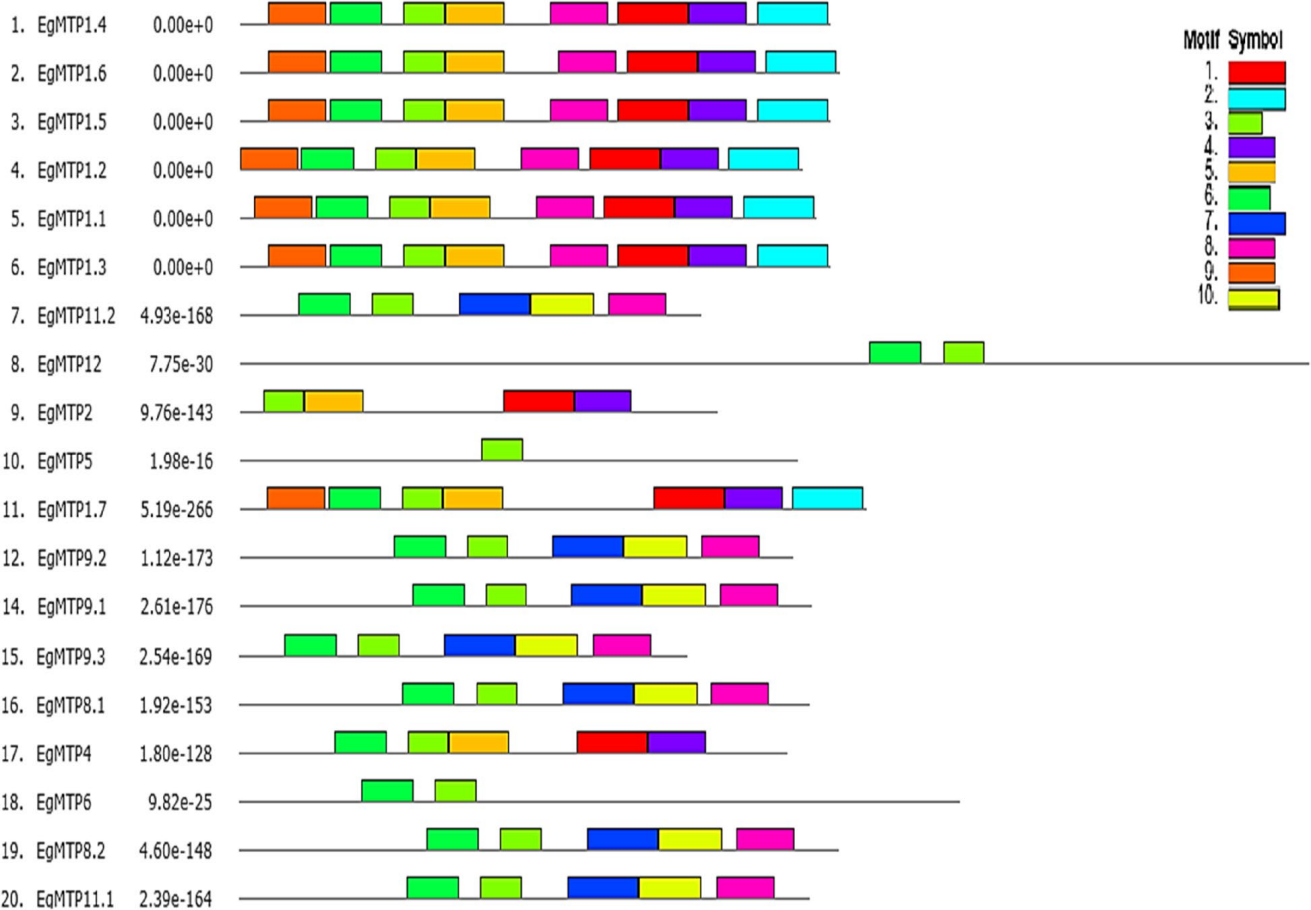
Conserved motifs were predicted by the MEME v.5.3.3 tool and displayed by the unique color mentioned in the box on the top right in different colored boxes. Among these represented motifs, four motifs possess specific functions related to MTPs. Motifs 1, 3, and 6 encode cation efflux domain and motif 8 encodes ZT dimerization domain

It was predicted that* EgMTP* proteins comprise 10 conserved sequence motifs in total, among which the *EgMTP*s contain only four motifs (1, 3, 6, and 8). They were annotated to Pfam database to have cation efflux function as six transmembrane domains (TMDs) and C-terminal ions transport domain (CTD) using the MEME tool (Fig. 4). Figure 4 reveals that the type and specific distribution of conserve motifs are different in the EgMTP proteins. Three motifs (1, 3, and 6) were shared by 19 *EgMTPs* proteins belonging to the cation efflux domain (cation_efflux; PF01545), while the other 13 members of *EgMTP* proteins contained motif 8 encoded ZT_dimer, PF16916 (the zinc transporter dimerization domain). All the *EgMTP*s were members of the Mn-MTP sub-family, composed of eight motifs with zinc transporter dimerization function Also, the MTP1.1 to MTP1.6 members of the *Zn-MTP* sub-family included a ZT dimer which contained a Zn transporter dimerization domain. The greatest number of the motifs (eight motifs) were found in MTP1.1 to MTP1.6, which also contained three CDF and Zn transporter dimerization domains, while MTP5 had only one motif.

### *Cis*-acting element analyses and the miRNAs target sites of *EgMTP* genes

To better understand the post-transcriptional mechanism of *EgMTP* genes, a search for *cis*-elements related to different stress responsiveness in the 1.5 kb of 5'-flanking region of most *EgMTP* genes was also observed by the PlantCare program (Supplementary Table S1). The promoter analyses identified 42 different *cis*-regulatory elements (CREs) in the upstream regions of whole *EgMTP* genes, which were divided into four main categories based on the regulation of gene expression in response to different factors, i.e. including several *cis*-elements in core promoter regions and light-, developmental-, hormone-, and environmental stress-responsive *cis*-elements (Supplementary Table S1). The results showed that the largest and smallest categories of CREs were light-regulated and developmentally regulated groups, respectively. The frequency of core *cis*-regulatory elements such as CAAT boxes and TATA boxes in all of the *EgMTP1.1* gene family is very diverse. *EgMTP6* contained nine CREs, which is the lowest number of CREs recorded in *EgMTP11.2*, however, it possessed forty-two CREs, which is the highest number of CREs and includes almost all the predicted regulatory *cis*-elements in *EgMTP* genes. Most *cis*-acting regulatory elements in the promoters of *EgMTP* genes family related to the response to light were frequently identified, and all of them are displayed in yellow in Supplementary Table S1. Moreover, the *cis*-regulatory elements related to hormone responsiveness were found upstream in the *EgMTP* gene promoter. The *cis*-regulatory elements related to MeJA responsiveness, including CGTCA and TGACG motif and abscisic acid-responsive element (ABRE), except for *EgMTP*1.2 and *EgMTP*6, were more abundant than the other phytohormone-responsive elements. Most of the *cis*-acting elements in the Mn-MTP sub-family were phytohormone-responsive, while the elements in the Zn/Fe-MTP and Zn-MTP sub-families were light-regulated. A few *cis*-active motifs, including CAT-box, HD-Zip 1, and GCN4, participated in the regulation of the developmental process in a number of *EgMTP* genes. Different stress-responsive CREs, i.e. circadian, ARE, MBS, TC-rich repeat, GC-motif, LTR, and O_2_ site were also identified in most *EgMTP* gene promoters. (Putative low-temperature responsive element (LTRE), anaerobic response elements (ARE), and O_2_ site (involved in the O_2_ regulation) were found more abundant than other stress-responsive CREs. Circadian (diurnal rhythm regulatory element), MBS (MYB binding site involved in drought-inducibility and flavonoid biosynthesis), TC-rich repeats (anaerobic response elements), and GC-motif (oxidative responsive element) were also other stress-responsive CREs in *EgMTP* genes. Totally, four hundred and twenty-five stress-responsive CREs were found in all *EgMTP* genes, indicating that the expression rate of *EgMTP* genes at mRNA level may be possibility affected by various stresses.

**Fig. 5:**
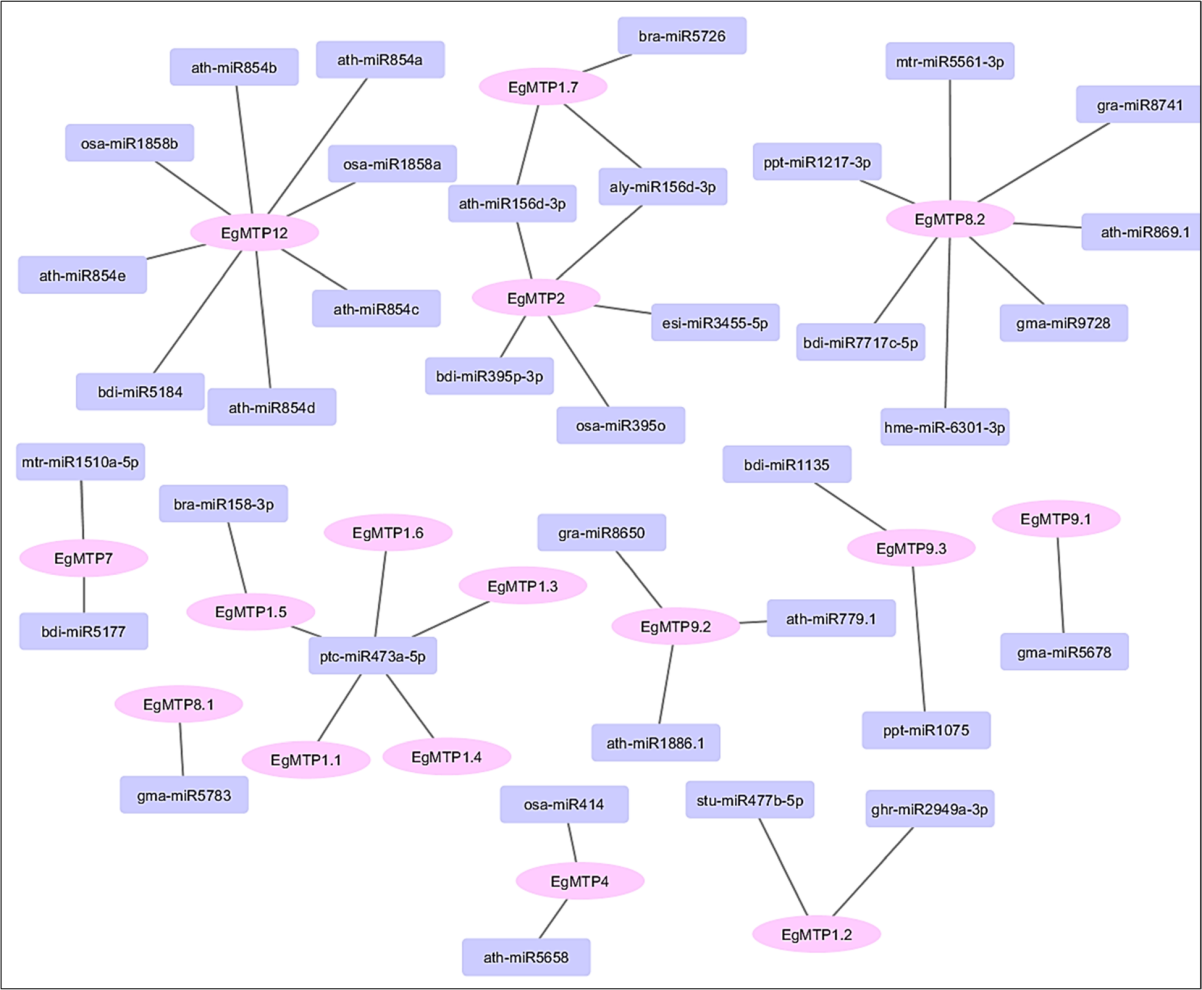
Interaction network of *EgMTP*-target miRNAs. Thirty-six miRNAs are targeted for the fourteen *EgMTP* genes. The genes are marked in pink and miRNAs in violet

The results of *EgMTP* coding sequence-based miRNAs showed a total of 36 miRNAs and 14 *EgMTPs* were predicted to be the targets for cleavage inhibition (Fig. 5). All *EgMTP* genes were targeted by more than one miRNA, while *EgMTP8.1* and *EgMTP9*.1 were suppressed by gma-miR5783 and gma-miR5678, respectively. *EgMTP12* and *EgMTP8.2* were targets for eight and seven individual miRNAs, respectively; ath-miR854b, ath-miR854a, ath-miR854e, ath-miR854c, osa-miR1858a, osa-miR1858b, and bdi-miR5184 for EgMTP12, and ath-miR869.1, gra-miR8741, gma-miR-6301-3p, hme-miR-6301-3p, bdi-miR7717c-5p, ppt-miR1217-3p, and mtr-miR5561-3P for *EgMTP8.2*. The interaction network of *EgMTP*-target miRNAs showed that *EgMT1.1, EgMTP1.4, EgMTP1.3, EgMTP1.6,* and *EgMTP1.5* contained the target site of ptc-miR473a-5p, however, *EgMTP1.5* was targeted by two other miRNAs, bra-miR158-3p.

### Gene ontology annotation (GO) and codon usage pattern (CUP)

**Fig. 6:**
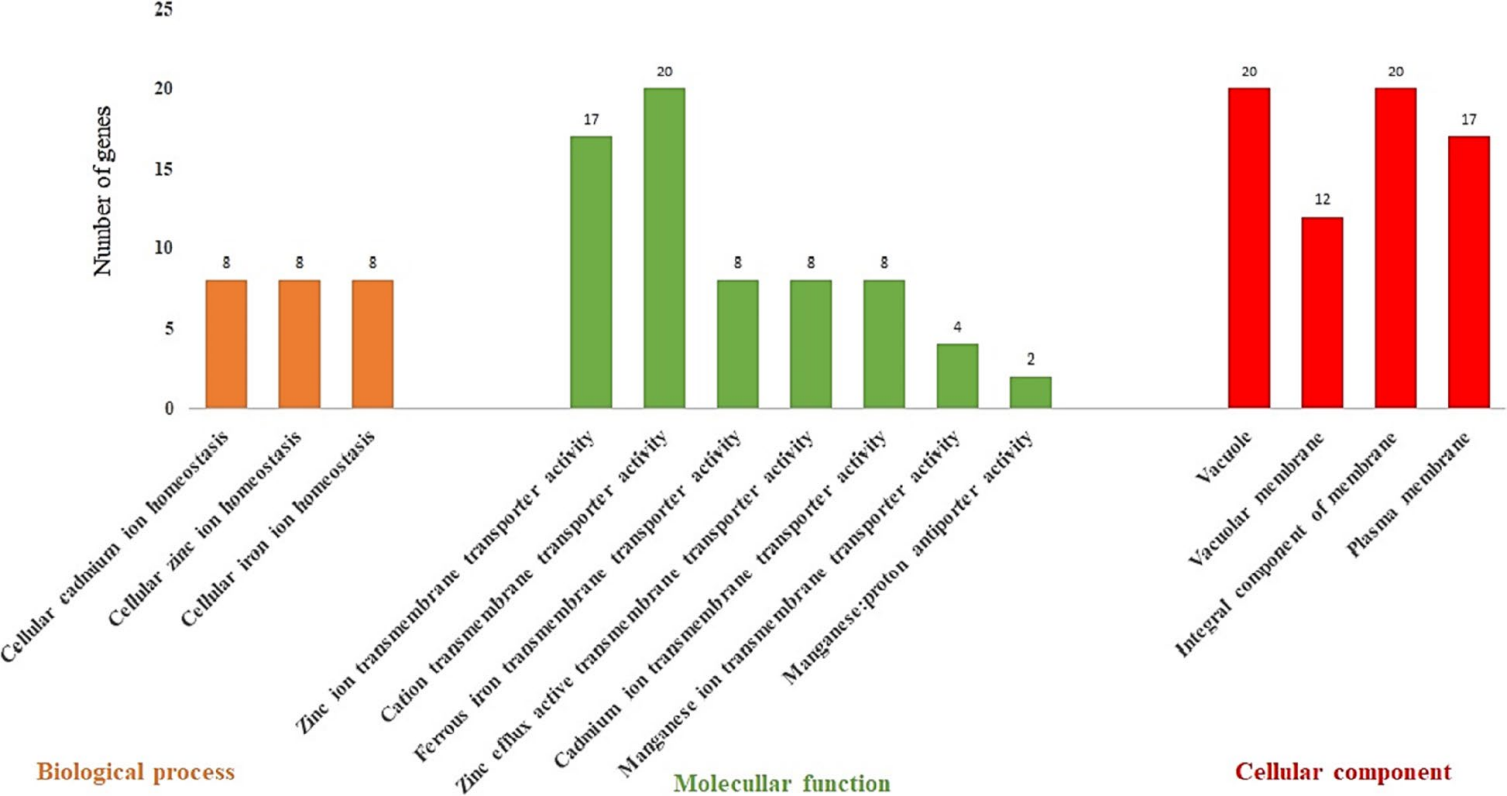
Gene ontology analysis of *EgMTP* genes. The analysis shows biological processes, molecular function, and cellular localization of the *EgMTP* genes

**Fig. 7:**
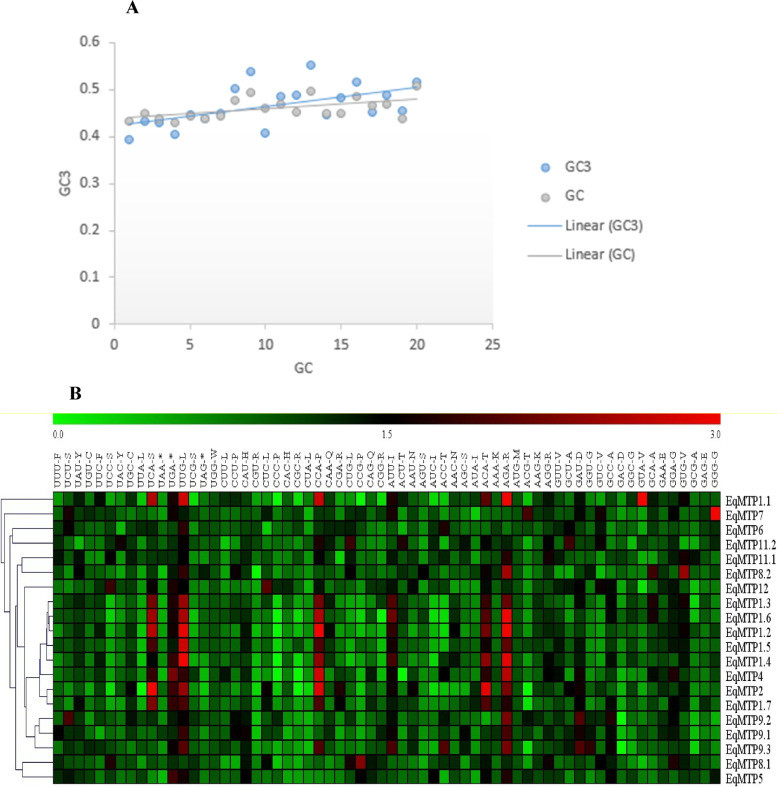
**A** The correlation between GC and GC3 contents in *EgMTP* genes. **B** The heat map of the relative synonymous codon usage analysis (RSCU) values of *EgMTP*s. The color boxes indicate RSCU values, the lowest (green) and the highest (red) codons usage. Green color indicates RSCU < 1 and dark red and distinct red represent RSCU > 1 and RSCU > 1.6, respectively

The subcellular localization, molecular function, and biological process of the 20 *EgMTP*s were predicted using GO enrichment analysis by STRING software (Fig. 6). The maximum scores of EgMTP proteins distribution in the cellular component were 20/100% in the vacuole and integral component members and then 17/85% in the vacuolar member. Our analysis of the molecular function of 20 *EgMTP*s exhibited a significant activity (20/100%) in the cation transmembrane transporter and zinc ion transmembrane transporter (17/85%). The total scores of MTP protein functions in various biological workflows were 8/40% in cellular cadmium, 8/40% in zinc, and 8/40% in iron homeostasis. Diverse genetic indexes such as ENC, RSCU, CBI, SChi2, etc. (Table 3) were used to investigate the codon usage pattern. The results showed that the GC values for *EgMTP*s were spanned from lowest to the highest values of 0.430 and 0.508 in *EgMTP1.1* and *EgMTP5*, respectively*.* The GC content at the third codon position value ranged from 0.392 to 0.538, which were observed in *EgMTP1.6* and *EgMTP12*, respectively. The overall analysis revealed no possibility of a direct correlation between GC and GC3 content. This correlation demonstrated that the mutation acts as the main factor in codon formation (Fig. 7 A), natural selection codon formation can be associated with the lack of correlation between GC and GC3s [30]. The ENC value was the lowest, ranging from 47.68 to 60.26 in *EgMTP2* and *EgMTP5, respectively,* indicating that one codon was used to proceed further. However, the highest one illustrated that all the same amino acid codons were used in the same amount to code an amino acid [31]. Codon usage bias is measured by CBI, the directional measurement index, and its value ranges from 0 to 1. In the present study, CBI was in the range of 0.153 to 0.395, which was observed in *EgMTP6* and *EgMTP2*, respectively. The frequency of RSCU in most of the 20 genes of *EgMTP* was in the range of 0 to 3, and the genes with the RSCU were equal to zero, such as *EgMTP4* (CGC-R, ACU-T, CCC-P), *EgMTP2* (UUA-T, CGC-R, UUA-L), *EgMTP1.6* (CCC-P, CGG-R), *EgMTP8.1* (CGU-R), *EgMTP1.4, EgMTP1.2, EgMTP1.1* (CCC-P), *EgMTP9.2,* and *EgMTP9.3* (GAC-D). Finally, the codon usage pattern of *EgMTP* genes was exhibited by the heatmap analysis (Fig. 7 B). The various colors displayed in the heatmap represent the over-represented and under-represented codons in *EgMTP* genes. SChi2 estimated the discrepancy between the number of codons observed and the number of codons anticipated from the equal use of codons. The SChi2 mean values of these genes were low, ranging from 0.08 to 0.46 in *EgMTP5* and *EgMTP2*, which shows a contrasting result with the highest and the lowest ENC values. SChi2 with a high value indicated a greater difference from the casual utilization of the synonymous codons [32].

**Table 3:** The codon usage pattern (CUP) in the *EgMTP*s gene of *E. grandis* was investigated using several genetics

Gene Name	ENC	GC3	GC2	GC	CBI	SChi2
*EgMTP1.6*	49.441	0.392	0.47	0.433	0.336	0.347
*EgMTP1.3*	53.233	0.433	0.474	0.45	0.305	0.236
*EgMTP1.2*	48.449	0.429	0.466	0.439	0.386	0.401
*EgMTP1.1*	49.133	0.404	0.473	0.43	0.369	0.417
*EgMTP1.5*	55.974	0.447	0.475	0.444	0.255	0.193
*EgMTP1.4*	50.4	0.438	0.478	0.439	0.361	0.377
*EgMTP4*	54.152	0.45	0.475	0.442	0.266	0.205
*EqMTP11.1*	59.122	0.502	0.486	0.476	0.186	0.11
*EgMTP12*	55.462	0.538	0.473	0.493	0.247	0.171
*EgMTP2*	47.68	0.406	0.523	0.46	0.395	0.496
*EgMTP6*	60.321	0.484	0.464	0.469	0.153	0.093
*EgMTP9.2*	57.278	0.487	0.449	0.453	0.204	0.141
*EgMTP8.1*	57.836	0.552	0.511	0.497	0.211	0.14
*EgMTP9.1*	58.071	0.447	0.46	0.45	0.244	0.147
*EgMTP8.2*	56.085	0.481	0.422	0.45	0.217	0.166
*EgMTP7*	60.036	0.515	0.434	0.486	0.168	0.101
*EgMTP1.7*	54.56	0.452	0.479	0.466	0.262	0.178
*EgMTP11.2*	59.731	0.489	0.466	0.468	0.208	0.137
*EgMTP9.3*	55.074	0.454	0.467	0.439	0.273	0.205
*EgMTP5*	60.262	0.516	0.521	0.508	0.174	0.089

The sequences were evaluated for GC content, GC content at the second codon positions (GC2), GC content at the third site position (GC3s), codon bias index (CBI), effective codon number (ENC), and scaled chi-square (SChi2)

ENC (demonstrating between 21- 61); GC < 0.5 (meaning no perceptible preference for GC nucleotides); GC3s < 0.5 (indicating the preference of the codons end with A/T); CBI (determining the use of synonymous codons, 0 meaning the use of synonymous codons, and 1 meaning maximum codon bias); SChi2 (showing deviation from the random use of synonymous codons)

### SSR markers

The analysis of SSR markers showed that the 57 SSRs were predicted in 18 *EgMTP* sequences (Table 4). Almost most of these genes, including *EgMTP1.4, EgMTP1.6, EgMTP1.1, EgMTP1.2, EgMTP9.1, EgMTP6, EgMTP8.2, and EgMTP11.1,* had a single SSR. Yet, *EgMTP1.3* and *EgMTP4* contained two SSRs with (CAT)4-(AAAT)3 and (ATC)4-(CAT)4 sequences, respectively. Among them, *EgMTP1.7* and *EgMTP5* contained seven SSRs; (TTG)4- (AATT)4- (ATTT)3- (AATT)3- (TTTA)3-(TGATG)3-(GGTCAC)3 and (CT)8-(TG)8-(AAG)4-(TCA)4-(AAAG)3- (TTAT)3-(GAGCA)3, respectively. Three SSRs were identified in *EgMTP12* and *EgMTP4* and four and five SSRs were predicted in *EgMTP9.3* and *EgMTP8.1*, respectively. The highest SSR type belonged to the tri-nucleotide SSRs with 22SSRs, followed by di-nucleotide SSR with 13 SSRs, tetra-nucleotide SSR with 20 SSRs, and penta-nucleotide SSR with 3 SSRs.

**Table 4:** Fifty-seven SSRs (simple sequence repeat) identified in eighteen of twenty *MTP* sequences in *Eucalyptus grandis*

Gene Name	SSR Motifs
*EgMTP1.4*	(CAT)4
*EgMTP1.6*	(CAT)4
*EgMTP1.2*	(CAT)4
*EgMTP1.1*	(CAT)4
*EgMTP1.3*	(CAT)4-(AAAT)3
*EgMTP12*	(GGC)6-(CAT)4- (TTCCCA)4
*EgMTP5*	(CT)8-(TG)8-(AAG)4-(TCA)4-(AAAG)3- (TTAT)3-(GAGCA)3
*EgMTP1.7*	(TTG)4- (AATT)4- (ATTT)3- (AATT)3- (TTTA)3-(TGATG)3-(GGTCAC)3
*EgMTP9.1*	(GAC)4
*EgMTP9.3*	(ACC)6-(TGTA)9-(ATGG)4-(GAAAAA)3
*EgMTP4*	(ATC)4-(CAT)4
*EgMTP6*	(TTTA)3
*EgMTP8.2*	(CT)12
*EgMTP1.5*	(CGC)6- (CAT)4- (TTTC)5
*EgMTP9.2*	(TC)20- (GA)19-(CGA)4-(TTTTC)3
*EgMTP7*	(CT)7-(GA)15-(CT)16- (TC)15-(CGA)7-(CTC)4-(CGA)7-(ATT)6-(GATG)3- (CTCC)4-(TTTTA)3
*EgMTP8.1*	(TC)12-(TC)10-(GA)11- (GAG)4- (TCCGT)4
*EgMTP11.1*	(TC)9

### *In silico* expression profile analysis of *EgMTP* genes in different tissues

**Fig. 8:**
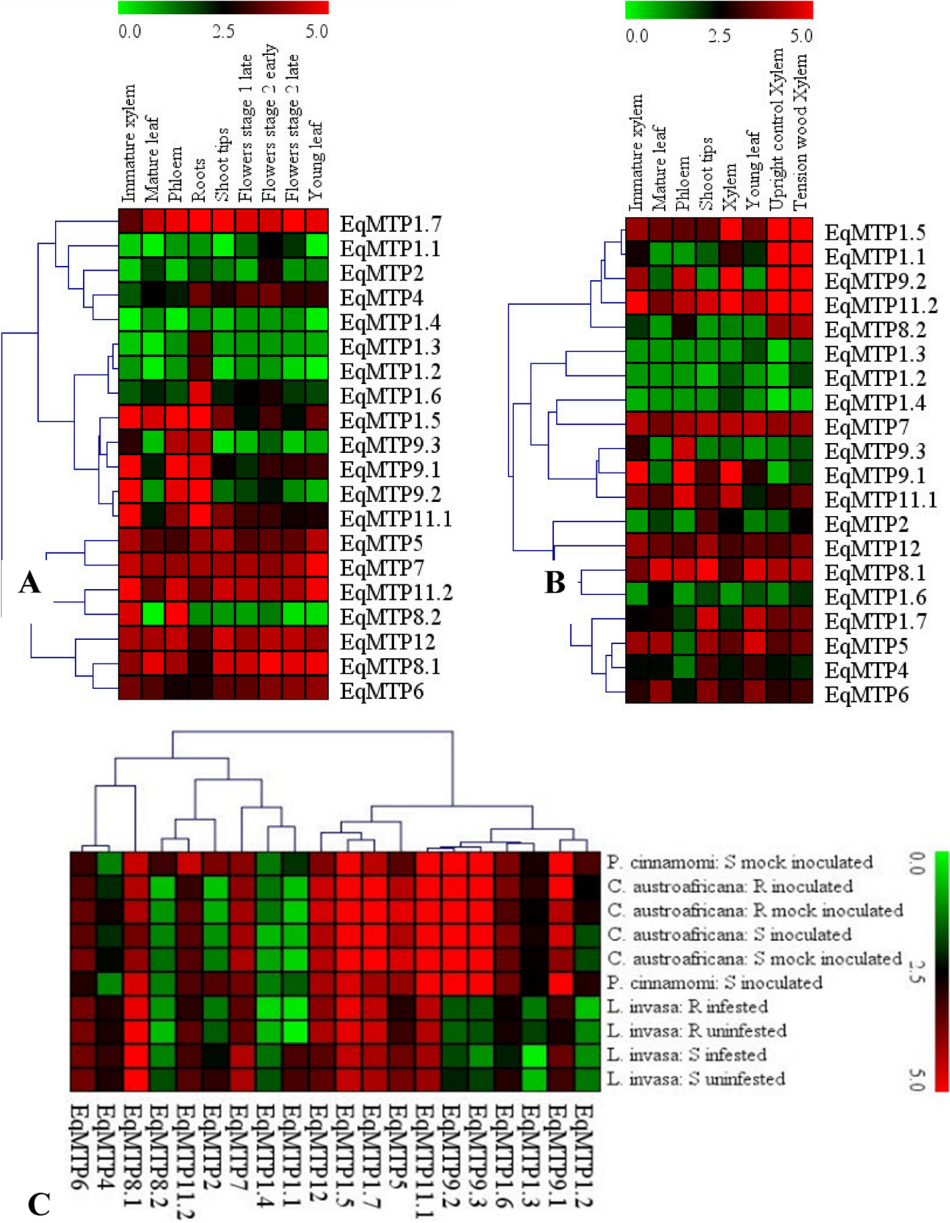
A gene expression heat map diagram of all *MTP* genes. **A** *E. grandis* TAGoo14 clonal genotype in six tissue samples, e.g. immature xylem, phloem, young leaves, mature leaves, shoot tips, roots, and flowers. **B** F1 hybrid of *E.grandis* × *E. urophylla* (GUSAP1clonal genotype) in immature xylem, mature xylem, phloem, young leaves, mature leaves, and shoot tips in addition to wood xylem and upright control xylem (cambium)*.* **C** Unsusceptible and resistant *E. nitens* seedling stems were inoculated with *Phytophthora cinnamomi* establishing and in mock-inoculated (control), sensitive four-month *E. Grandis* was split into uninfected and infested with *Leptocybe invasa,* and stems of one-year-old susceptible and resistant* E. grandis* were inoculated with *Chrysoporthe austroafricana.* The genes with similar profiles are grouped in hierarchical clustering. The intensity of expression is shown in the color bar, the highest (red) to lowest (green). The scale bar represents the log_2_ of TPM

A gene expression heat map diagram of all *MTP* genes of F1 hybrid *E. grandis* × *E. urophylla* (GUSAP1 clonal genotype) and *E. grandis* TAGoo14 clonal genotype in different tissue samples was drawn (Fig. 8 A and B). Comparing all *EgMTPs* with one another, the highest gene expression of *EgMTP* was related to *EgMTP11.2*, and the lowest gene expression belonged to *EgMTP1.3* / *EgMTP1.4* belonging to Mn and Zn subfamilies, respectively. More importantly, the abundance of *EgMTP* transcripts found in the young leaves of two compared species showed the vital role of *EgMTP* genes in the plant leaves and their potential to detoxify the elements. This conclusion agrees with the results obtained from previous reports, in which the metals transferred from the terrestrial parts to the aerial parts were observed in proteins with the nature of transferring metals. The increasing and decreasing trends in the expression of *EgMTP*s in tension wood xylem and upright control xylem were identical, and all *EgMTP*s increased in tension with wood xylem compared to the upright control xylem. The highest level of expression was also observed in *EgMTP11.2*, and the lowest level of expression was seen in *EgMTP 1.3* and *EgMTP 1.4*. Higher and lower levels of the similar gene in various tissues and species can contribute to the application of in silico data for selecting a more effective gene for in vivo experiments.

### Digital biotic gene expression

In this study, RNA-Seq data from the three types of biotic stresses were examined for different *Eucalyptus* species. The results obtained from EucGenIE RNA-Seq data exhibited the upregulation and downregulation patterns of *EgMTP* genes in the three biotic stresses, and the observed patterns were similar (Fig. 8C). However, the results of *EgMTP* genes expressions in the resistance and control samples (mock-inoculated) of inoculated *E. grandis* with *C*.* austroafricana* showed a higher *EgMTP* gene expression in control plants. It was concluded that the EgMTP family members were not essentially affected by *C*.* austroafricana,* whereas, the positive expression correlation of* EgMTP*s was observed in the stems of *E. nitens* inoculated with *P. cinnamomi.* When two biotic conditions were mentioned, the highest gene expression was observed in *EgMTP11.2* and the lowest gene expression was seen in *EgMTP1.4* and *EgMTP1.3*. Also, in comparison with the resistant genotype, the stems of susceptible *E. grandis* in the samples seem to be uninfected and infested with *L*. *invasa*, which reveals a higher gene expression. This demonstrated the negative correlation of the *EgMTP* genes expression with gall wasp caused by *L. invasa.*

### Transcription profile of *EgMTP* genes in response to heavy metal treatment

**Fig. 9:**
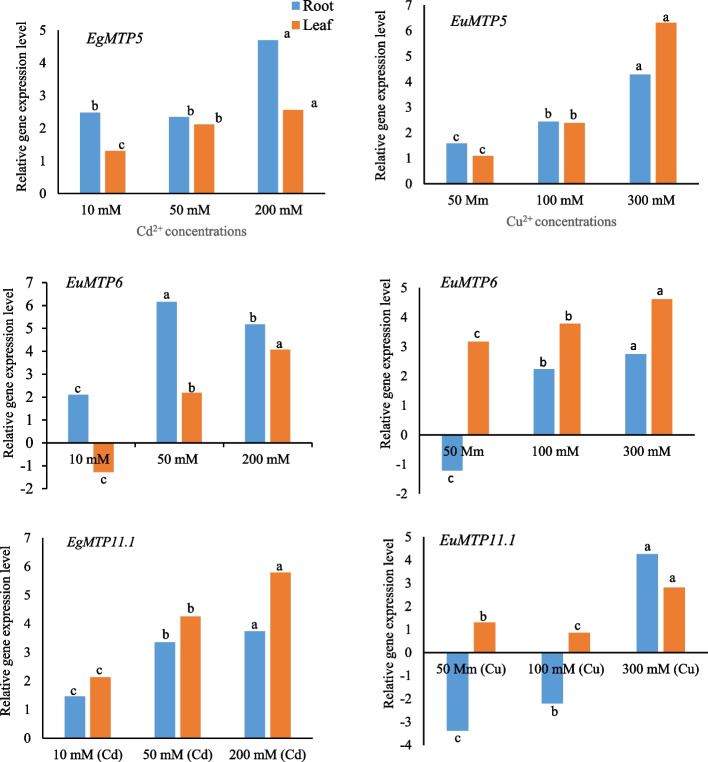
Relative expression analysis of *EgMTP5, EgMTP6,* and* EgMTP11* genes in the roots and leave of two-month *E. garndis* seedling exposed to 18-day excess metal by Cd(NO_3_)_2_ (10, 50, 200 mM) and CuSO_4_. 5H_2_O (50, 100, 300 mM). Different letters (a, b, c) indicated above the bar represent statistically significant difference at *p* ≤ 0.05 (Duncan's multiple range test)

To get an initial insight into *EgMTP* genes regulation in response to excessive heavy metal treatment stress, the relative expression of the three genes *EgMTP5*, *EgMTP6*, and *EgMTP11.1* at different concentrations of CuSO_4_ and Cd (NO_3_) in root and leaf tissues was investigated using qRT-PCR analysis (Fig. 9). On the subject of two different tissues, the transcript amounts of the three genes seem to be positively excessive heavy metal stress-dependent. As Fig. 9 shows, the relative expression ratios of *EgMTP5*, *EgMTP6*, and *EgMTP11.1* gradually upregulated at concentration of 50 mM CuSO_4_ (C1; 1.06-, 3.21-, and 1.29- fold, respectively) and steadily incremented at 100 mM (C2; 2.5- and 3.87-, and 0.98-fold, respectively), followed by boosting excessive concentration level at 300 mM (C3; 6.23-, 4.25, and 2.63-folds, respectively) in the treated leaves. With regard to the root tissue, the highest frequencies of *EgMTP5*, *EgMTP6*, and *EgMTP11.1* transcripts were observed under C3 circumstances (4.29-, 2.89-, 4.25-fold), while at lower levels were achieved C1 (2.36, -1.11, -3.42-fold) and C2 (1.15-, 2.35-, and -2.13-fold), respectively (Fig. 9). Subsequently, the same applies to the high concentration of Cd (NO_3_) treatment as Cu^2+^ stress, as the transcript amounts in *EgMTP5*, *EgMTP6*, and *EgMTP11.1* gradually increased, showing the peak values of ~ 2.91, 4.02, and 5.95 until C3, respectively.

According to the results, the greater the excessive heavy metal stress, the higher the transcript abundance of *EgMTP5*, *EgMTP6*, and *EgMTP11.1* genes, and vice versa.

## Discussion

Plant metal tolerance proteins are membrane divalent transporters that play a substantial role in the specific transport of different types of heavy metal ions as part of the response-specific metal toxicity. In addition to their pivotal function in maintaining mineral plants, changes in their transcript amounts can be considered environmental biomarkers in examining the heavy metal pollution level. The identification of *MTP* gene family has previously been reported in diverse plant species, including *Medicago truncatula* [33], *N. tabacum* [34], *V. vinifera* [21], *P. trichocarpa* [18], *Arachis hypogaea* [19], and *Solanum lycopersicum* [35]. However, there is no information specifically focusing on the genome-wide identification of the *MTP* family in *E. grandis* and concurrent assessment of the effect of different concentrations of heavy metals on the transcript amounts of the genes known to be expressed in response to metal stress.

Herein, 20 putative *EgMTP* genes were determined, selected, and nominated according to both their respective orthologous relationship and high similarities with the *Arabidopsis* MTP families. The diversity of physicochemical characteristics of the *EgMTP* genes, including length of CDS, molecular weight, pI, and subcellular localization, is consistent with previous reports demonstrating that the *MTP* gene family members play a role in several pathways and biochemical networks leading to resistance against metal stress [17, 28, 33, 36]. According to the results of the same study, all *EgMTPs* were localized in the vacuole membrane, so it could be concluded that *EgMTP* might act as cation transporters [35].

Our findings from phylogenetic analysis were in line with the previous results obtained from various plant species, suggesting that *EgMTPs* have a close evolutionary relationship with other plant species based on amino acid similarities and may have the same tasks [15, 18, 19, 34, 37]. Moreover, all *EgMTP* gene family members have orthologous relationships with *AtMTPs*, except *AtMTP3* (Fig. 1). Our results indicated that two phenomena, gene expansion or gene loss, occur during the evolution of MTP genes and the formation their members in various species [35].

Our results showed that all these *MTP* gene members clustered in seven groups (1–4, 5, 6, 7, 8, 9, and 12), in which about eleven, seven, and two *MTPs* belong to three major substrate-specific sub-families, i.e. Zn-MTPs, Mn-MTPs, and Zn/Fe-MTPs, respectively (Fig. 1), indicating the significance of evolutionary relationships in the deduction of structural and functional functions among species. Regarding phylogenetic relationships between MTPs genes and the three main groups, all the Mn-MTP sub-family genes had more than one homologous repeat, and only *EgMTP1* genes in the Zn-MTP group had more than one repeat. The genome-wide analysis revealed that *Vitis vinifera*, *Citrus sinensis*, and *Populus trichocarpa* contained more than one paralogous member of the Mn-MTP [18, 21, 28]. In addition, *EgMTP8* has been reported to have orthologous relationship with citrus, rice, maize, cucumber, and barley [3, 11, 16, 28].

In this study, to get more information about the gene annotation and expansion mechanism of the *EgMTP* gene family, the gene synteny and duplication were assessed (Fig. 2, Table 2). Two phenomena containing segmental and tandem duplication occur in two and more genes on different chromosomes and also in the same chromosomes, respectively [38]. The results showed that the *EgMTP* gene family members were distributed over nine chromosomes, in which the greatest number of *EgMTP* genes was detected on chromosomes 05 and 06 with 6 and 4 genes, respectively. Furthermore, the *EgMTP* genes contained two segmental duplication pairs, whereas 15 tandem duplications occurred, indicating the extension of *EgMTP* genes in *Eucalyptus* could be mostly ascribed to tandem duplications. The results revealed that nearly all *EgMTP* genes exposed gene duplication phenomena, among which *EgMTP1.5/ EgMTP1.7* and *EgMTP8.1/ EgMTP8.2* have segmental duplication genes. These results indicate the probability of a direct correlation between duplicated gene pairs, which has been obtained from phylogenetic analysis. The Ka/Ks ratio value of two types of gene duplication pairs was less than 1, suggesting that positive selection and functional divergence might occur before duplication events in *E. grandis* genome [39]. Previous studies have shown that both duplication and divergence events may be considered standard features occurring in the genes encoding key enzymes in secondary metabolite pathways during the evolution process [40]. Both segmental and tandem duplications can alter the organization of gene families, and the diversity in the number of genes in the studied *EgMTP*s may be linked to the duplication events causing evolution. Previous results indicate the generation of new genes with various roles can be achieved by gene duplication events [41]. Due to the importance of types of duplication in *MTP* gene family evolution in various plant species, it is expected to be the subject of future research [18]. Considering the number of gene family members in different species and genome sizes, no significant relationship was observed between the genome size and the number of gene family members. Eucalyptus and populous had the greatest number of *MTP* gene family members compared to other tree and crop species, which could be due to their important role in soil remediation in lands irrigated with wastewater, thereby helping the plant growth, production of wood, and more absorption and retention of metals in their organs [42].

Exon–intron structure divergence was originally proposed to be important to perceive the phylogenetic relationships within the gene families and the evolutionary history of gene duplication occurrences, thereby providing additional evidence [43–45]. Based on the results, it can be deduced that there is a remarkable correlation between the structure of exon/intron and phylogeny among *EgMTPs* (Fig. 3). Our results showed the maximum association between the sub-families and the maximum structural diversity between the *EgMTP* sub-families. Exon–intron structures revealed that a negative selection was also detectable in all the *EgMTP*s, showing that the new functions in the family members were caused by substitution or divergence in the exon–intron structures [43]. In addition to the repeated copies, all the Mn-MTP sub-families revealed an alternative splicing in the gene expression process, whereas only one gene of the other two sub-families had alternative splicing. Shirazi et al. [21] showed the same pattern of alternative splicing of Mn-MTP gene members and the exon–intron structures in *VvMTP* members [21]. As discussed earlier, maximum and minimum mean distances were observed in the Zn-MTP and Mn-MTP sub-families, respectively. Furthermore, maximum and minimum uniformities were observed in the Mn-MTP and Zn-MTP sub-families, respectively. Moreover, these two sub-families had the highest mean distances between them. The above-mentioned differences and similarities indicate the changes of the *EgMTP* genes sequences in the evolutionary relationship of the *EgMTP* genes. It seems that the existence of different copies of gene with the same functions in plants can be due to their high needs based on gene specific roles or different expressions in organs, developmental stages, and different stress conditions.

The maximum numbers of the *EgMTP* proteins comprised four to six recognized transmembrane domains (TMDs), which go along with the report of prior research reporting similar results for the MTP families in various plant species [14, 46, 47], except for *EgMT12* with 12 TMDs, including CitMTP12 [28]. The results indicated all twenty *EgMTPs* were predicted to be localized in the vacuoles as primary sites for various processes like detoxification, stopping, and storage of metals. This result was validated by previous studies, demonstrating the *VvMTP* and *TaMTP* members were localized in the vacuoles [17, 21]. Although the distribution of conserve motifs varied specifically among *EgMTP* proteins, overall, the numbers of cation efflux domain were highest in the 19 *EgMTPs* proteins, while 13 members of the *EgMTP* proteins contained specific motifs encoding the zinc transporter dimerization domain (Fig. 4). The analysis of conserved motifs showed that the *EgMTPs* belong to the Mn-MTP sub-family, and the MTP1.1 to MTP1.6 members of the Zn-MTP sub-family contain motifs with detoxification of Mn and zinc transporter dimerization, respectively. Previous studies have reported that various protein domains can play important roles in determining the functions of proteins, consequently, they might have a pivotal role in the evolution rate. Kobae et al. [48] reported that AtMTP1 plays a role in the transport of excess Zn into the vacuoles, resulting in the control of Zn homeostasis in cells. In addition,, Arrivault et al. showed the role of AtMTP3 in the detoxification of Zn and/or Co [49]. Migocka et al. [11] proposed that Zn transporters could act as metal ion transporters that assist the detoxification of metals in the vacuoles. The ZT-dimerization domain has also been recognized with the functions of the dimerization of ion transporters, causing the establishment of the homodimers or heterodimers of MTPs during Zn transport [46]. It is believed that Mn-MTPs are involved in transporting one or more substances [13]. Also, some researches have shown the Mn-MTPs containing the members of MTP8, MTP9, MTP10, and MTP11 have special functions in different plant species [11, 50–54]. CREs act as an essential component in regulating the transcription of the time-, location-, and environmental-responsive genes by interconnecting with special transcription factors and RNA polymerase [19, 55]. Our results indicated a large number of consensus sequences such as CAAT-box and TATA-box were displayed in the upstream regions of *EgMTP* genes mainly involved in the control of expression rate and initiation of transcription. Besides, the promoter regions of *EgMTP* genes containing a wide range of special elements responding to light, phytohormone, and abiotic stresses present in the 5' flanking region of *EgMTP* genes (Supplementary Table S1) indicate that the transcription rate of *EgMTP* genes can be modulated by TF response to different stimuli in multiple pathways. Most *cis*-acting elements in the *EgMTP* genes were found to respond to light, and many of them were also reported in the promoter regions of MTP gene family members of grape and wheat [11, 21]. Hence, we can conclude that these special *CREs* in the 5’-flanking regions of the *EgMTPs* play an essential role in the regulation of *MTP* genes responding to light. Previous reports have indicated the light-dependent mechanisms in plants are complex and several developmental and physiological mechanisms like phototropism, flower initiation, and diurnal rhythms are affected by light [56]. Our findings showed the combination of substantial light responsive *cis* elements, indicating the function of light as an important modulator for the expression of* EgMTP* genes. Furthermore, our results showed the Mn-MTP sub-family genes, including EgMTP8.1, EgMTP8.2, EgMTP9.1, EgMTP9.2, EgMTP9.3, EgMTP11.1, and EgMTP11.2, harbor ABRE, where they were involved in response to the abscisic acid treatment and drought and salt stress in Arabidopsis [57]. AREs *cis*-elements are important in the induction of genes exposed to anaerobic conditions [58], and most abundant regulatory elements present in the *EgMTP* genes belong to the Zn/Fe-MTP and Zn-MTP sub-families.

MicroRNAs, small non-coding RNAs, generally perform a regulatory function as downregulating the transcript amounts of genes by not only cleavage specific target mRNA but also by preventing the translation the response of target genes [59, 60]. Several mechanisms related to several functions such as the plant growth and development, signal transduction, and response to heavy metal stress are believed to be controlled by miRNAs [61, 62]. Bioinformatic tools provide a beneficial way to study the potential of the miRNA interactions related to special gene families [63]. In the present study, a total of 36 miRNAs related to 14 *EgMTPs* were identified. Five pairs of *EgMTP* genes (*EgMTP1.1*, *EgMTP1.3*, *EgMTP1.4*, *EgMTP1.4*, *EgMTP1.5*, and *EgMTP1.6*) were predicted as miR473a-5p targets and were shown to regulate the genes in drought-responsive genes in *Populus* plants [64]. Besides, two pairs of *EgMTP* genes, including *EgMTP2* and *EgMTP1.7,* were suppressed by miR156, playing dominant roles in regulating plant development and detoxification of heavy metals, such as cadmium, aluminum, manganese, and arserinc in *Brassica napus*, *Oryza sativa*, *Glycine soja*, *Populus vulgaris*, and *Brassica junce*a [65–72], and regulating the plant juvenile-to-adult transition [73]. *EgMTP2* was also predicted as the target of miR395p-3p, regulating sulfate starvation and balancing the ion concentration in plants [63]. The above findings provide an accurate insight into the role of predicted miRNAs in the *Eucalyptus* genome and clarify their functions in regulating metal tolerance in *Eucalyptus* plants. Gene ontology is an essential analysis to anticipate the recognized subcellular localization, molecular function, and biological process in organisms [73]. The gene ontology results and conception can be utilized to describe the relationships and gene function. In the present study, gene ontology examination showed the substantial activity of *EgMTP* protein molecules in cation transmembrane transporter, especially zinc ion, which is suggestive of a substantial role in the transporting of heavy metals (Fig. 6). Furthermore, GO analysis revealed the molecular functions of *EgMTPs* participating in processes related to the activity of zinc, ferrous, cadmium, and manganese transmembrane transporters and in the biological process of cellular zinc, cadmium, and iron homeostasis. Further, *EgMTPs* were observed to be vacuole and integral components based on the cellular component results, which is also in line with the results obtained from TMD numbers with cytosolic N and C termini for predicting cellular localization, verifying the situation of *EgMTP*s in the vacuole of the *Eucalyptus*. It seems that *EgMTP* gene families are now believed to encode the membrane proteins in the transferring metals into the vacuole, which promotes the effectiveness of *Eucalyptus* phytoremediation.

The codon usage pattern (CUP) analysis was originally proposed to predict the evolutionary pattern of genome and is used to evaluate the accuracy or efficiency of translation. Our results showed the ENC indices with the values 57 to 59.7, suggesting that the synonymous codons were possibly utilized to code the amino acids of the EgMTPs belonging to the Mn-MTPs sub-family [31], while the *EgMTPs* in the Zn-MTPs comprise only one codon. In contrast, the SChi2 value of *EgMTP* genes in the *Zn-MTP*s was higher than that of the Mn-MTPs sub-family. The high value of SChi2 suggests a stronger divergence from the casual utilization of synonymous codons [47]. Most of the *EgMTP*s showed GC values of < 0.5, suggesting that these *EgMTP* genes had no appreciable priority over GC nucleotides. Moreover, most of them had GC3 values of < 0.5, demonstrating that the codons with A/T end were preferable. CBI index, a directional calculation of codon usage bias, determines the synonymous codons utilized in a gene. In this study, the CBI value was low, which was changed from 0 (similar utilization of synonymous codons) to 1 (maximum codon bias) [74].

SSRs, repetitive sequences of simple 1–6 nucleotide motifs, are essential elements that play a remarkable function in the modulation of transcription rate [75]. The major SSRs in diverse plant species were presumed to be diverse. For example, in monocots, except for maize, the main SSRs are CCG/CGG/CGC/GCG/GCC/GG, while in dicots, the dominant SSRs are AAT/ATT/ATA/TAT/TAA/TTA. The predominant SSR types are taxon-dependent, although the frequency of AT-type SSR is believed to be maximum in dicot plant species [76]. Our results reveled the highest SSR type in the *EgMTPs* were predicated to be tri-nucleotide SSRs with 22SSRs, followed by di-nucleotide SSR with 13 SSRs, tetra-nucleotide SSR with 20 SSRs, and penta-nucleotide SSR with 3 SSRs (Table 4). The diverse numbers of SSRs in the *EgMTP* gene sequences suggest that the pattern mentioned above are used to identify the polymorphism between the cultivars of different *Eucalyptus* species, determining their relationship with the potential of heavy metal tolerance and helping to identify more resistant cultivars based on the marker-assisted selection in the plant breeding progress.

*EgMTP* gene expression analysis based on the previously published RNA-seq data shows a different response to different developmental phases and biotic stresses in various *Eucalyptus* species (Fig. 8). Digital data indicate the genes in different tissues and species have identical expression patterns, contributing to understanding the use of in silico data in selecting a more effective gene for in vivo experiments. Studies on different species exhibit the maximum transcript accumulation of *EgMTP11.2* and *EgMTP11.1* take place during the developmental stage and biotic stress, respectively. It is also in line with the essential function of the variants of the* EgMTP11* gene in *Eucalyptus* plants and in vivo gene analysis. *EgMTP11* gene might be used as an excellent marker gene, as genes containing two important ion transport domains and one ZT-dimerization domain transport divalent cations from the cytosol into organelles. However, our results showed *EgMTP11.1* has a maximum number of CRE regulatory elements in the 5’-flanking region in response to light, phytohormone, environmental stress, and developmental stages. Moreover, the presence of multiple alternative splicing in the *EgMTP11.1* and *EgMTP11.2* may reflect differences in the roles of the respective genes in various conditions and developmental processes. Previous results show that *Eucalyptus* requires the expression of *EgMTP11*, which plays an essential role in Mn transportation and detoxification [50–52]. The possible function of *CsMTP* genes in *Citrus sinensis* was examined in the presence of excessive Cu^2+^ ions and *CsMTP11* significantly up-regulated under different excessive Cu^2+^ treatments [28]. The researchers suggested that *MTP3, MTP7, MTP11, MTP1*, and *MTP9* genes are all upregulated in plants under detrimental conditions, e.g. salinity, osmosis, and drought stresses [77]. Although toxicity with heavy metals can result in similar responses to salinity stress, it may suggest salinity stress results in the storage of excessive ions in plants and decreases the agricultural yields [78, 79].

Based on the results obtained above, most of *EgMTP* genes were predicted to be different under various conditions and developmental processes, as we speculated that the transcription activity of *EgMTP* genes might be affected due to the balancing of metal concentration in leaves and roots. To test this hypothesis, the transcript amounts of *EgMTP5*, *EgMTP6*, and *EgMTP11.1* in the root and leave tissues of one *E. grandis* cultivar was investigated at different concentrations of CuSO_4_ and Cd (NO_3_). For both leaves and roots, compared to the control, the transcription rate of the three genes slightly exhibited some positive alterations under excess Cu^2+^ and Cd^2+^ concentrations. In agreement with previous studies [33], we found that the transcript amounts of *EgMTPs* were more affected by metal ion exposure in the roots than in the leaves, suggesting its important role in the translocation of divalent metals. Previous results have shown that *SlMTP* genes exhibit distinctive responses in either plant leaves or roots in response to different concentrations of heavy metals. The upregulation of *EgMTP5*, *EgMTP6*, and *EgMTP11.1* might contribute to the Cu^+2^ and Cd^+2^ transposition via suppression of vacuolar and vesicular ions exposed to excessive metal. *SlMTP1*, *SlMTP3*, *SlMTP4*, *SlMTP8*, *SlMTP10,* and *SlMTP11* display maximum expression responses when exposed to different concentrations of heavy metals [35]. Previous studies have revealed the considerable role of MTP genes in increasing the toleration of plant under metal treatment [15, 18] by inducing the metal transporter from the cytoplasm to transport Zn^2+^, Ni^2+^, Co^2+^, Cd^2+^, Fe^2+^, and Mn^2+^ [3]. Furthermore, the high expression of MTPs can be utilized as a bio-environmental indication to anticipate the contamination of heavy metals based on their transcription amounts [33]. In conclusion, 20 *EgMTP* genes were determined in *Eucalyptus grandis*, which were phylogenetically clustered in seven groups (1–4, 5, 6, 7, 8, 9, and 12) as a property of three main substrate-specific clusters (Mn-MTPs, Zn/Fe-MTPs, and Zn-MTPs). It seems all *EgMTP* genes experience expansion and gene loss by gene duplication events, among which *EgMTP1.5/ EgMTP1.7* and *EgMTP8.1/ EgMTP8.2* might be segmentally duplicated genes. Moreover, we ascertained different CRE elements associated with light, phytohormone, and abiotic stresses in the 5’-flanking regions of *EgMTP* genes, indicating that they might take part in activated signaling pathway under different environmental stimuli. Also, a total of 36 miRNAs corresponding to 14 *EgMTPs* were identified, which play dominant roles in regulating plant development and heavy metal detoxification. Our results revealed that most of *EgMTPs* participated in processes related to heavy metal transportation and ion homeostasis. The transcription activity of *EgMTP5, EgMTP6,* and *EgMTP11* genes seems to be positively upregulated under excessive Cu^2+^ and Cd^2+^ concentrations. Our results showed the essential roles of EgMTP proteins in the absorption and translocations of heavy metals in Eucalyptus species, which could be useful in selecting a variant with high resistance to heavy metals and improving modified cultivars which act better in stressful environments in future.

## Methods

### Identification of *Eucalyptus MTP* genes

To identify *EgMTP* genes in *Eucalyptus grandis*, the protein sequences of *Arabidopsis thaliana* (AT2G46800.1, AT3G58810.1, AT2G29410.1, AT2G47830.1, AT2G04620.1, AT2G39450.1, AT1G79520.2, AT1G16310.1, AT1G51610.1, AT3G58060.1, AT3G12100.1, and AT3G61940.1) from the Arabidopsis Information Resource (http://.arabidopsis.org) [28] and *Oryza sativa* (Os05g38670, Os04g23180, Os02g58580, Os03g12530, Os01g62070, Os05g03780, Os02g53490, Os08g32650, Os01g03914, and Os03g22550) from Rice Genome Annotation Database (http://rice.plantbiology.msu.edu/) [17] were obtained, BLASTP at e-value of < 1e^−10^ was investigated via Phytozome database, and these sequences were used as queries to compare with the *Eucalyptus* genome [80]. All candidate MTP protein sequences were analyzed by the HMMScan tool (https://www.ebi.ac.uk/Tools/hmmer/search/hmmscan) for Hidden Markov Model (HMM) profiling of PF01545, the cation efflux domain, on the Pfam website. Finally, the HMMER-EMBL-EBI database was used to confirm the predicted domain [29].

### Phylogenetic analysis and nomenclature

The MTP protein sequences of *Eucalyptus*, *Populus*, *Arabidopsis*, and *Oryza* were aligned by ClustalX 2.0.8 with default parameters [81]. To display the evolutionary MTP relationships, a phylogenetic tree was made by the MEGA 5.2 program using the neighbor-joining (NJ) method with 1000 bootstrap replicates [82]. The identified *MTP* gene family from* Eucalyptus* was nominated as *EgMTP* based on sequence similarities, and Matrix Global Alignment Tool (MatGAT) software was used to confirm the evolutionary relationship with *AtMTPs* and *OsMTP* in Arabidopsis and Oryza, respectively [83].

### Sequence analysis of the MTP proteins

The physiochemical features of MTP family protein sequences were analyzed to obtain molecular weights (kDa), isoelectric points (pI), etc. by ProtParam tool (http://web.expasy.org/protparam) [84]. Furthermore, the subcellular location of protein sequences for EgMTP were predicted by the Plant-mPLoc [85]. TMD, motifs as transmembrane helix, was also recognized using the TMHMM server, v.2.0 [86]. Also, the conserved gene family motifs were examined using the MEME v.5.3.3 tool by altering the appendix parameters: 60 ≥ width ≥ 5 and 10 motifs as the maximum number [87]. The functional potential of these motifs was assessed by the HMMScan tool.

### Chromosomal location, duplication, and selection pressure

The *Eucalyptus* genetic database was utilized to identify the location of the *EgMTP* genes on chromosomes, which were used to create a location image, as linkage groups on different chromosomes using TBtools genetic mapping software. Furthermore, the plant genome duplication database (PGDD) was analyzed to identify segmental duplication. Tandem duplication was considered with the following parameters: maximum distance 20 genes with > 75% coverage and > 75% similarity in aligned sequences [20, 88, 89]. The Ka/Ks ratios (the number of nonsynonymous site (Ka) and the number of synonymous sites (Ks)) were calculated by a simple Ka/Ks calculator using the DnaSP program. The Ka / Ks = 1, Ka / Ks > 1 and Ka / Ks < 1 ratios indicated natural selection, positive selection and purifying selection, respectively [90]. The probability of substitution (r) from one base to another was estimated based on the model presented by Tamura et al. [91] along with the maximum likelihood method and computed between and within the groups using the MEGA software [92].

### Gene structure, promoter analysis, simple sequence repeat (SSR) markers and microRNA target sites of *EgMTP* genes

The structural analysis of the predicted *EgMTP* genes was carried out to investigate the organization of intron/exon and the number of exons and introns in these genes using both sequences of gDNA and CDS by the gene structure display server (GSDS) [93]. All *cis*-acting regulatory elements (CREs) of the *EgMTP* gene promoter were identified using PlantCARE database (http://www.bioinformatics.psb. ugent.be/webtools/plantcare/html/) [94]. Also, the numbers and sequence types of SSR markers were predicted in the *EgMTP* gene family using the BatchPrimer3v1.0 server [95]. miRNAs target sites in the coding sequences of genes were analyzed by the psRNATarget database using the appendix factors: max expectation 3 and target accessibility (UPE) 25, and Cytoscapee software was utilized to predict the miRNAs [96].

### Gene ontology annotation (GO) and codon usage pattern (CUP)

All identified EgMTP protein sequences were used to analyze GO annotation according to all the three GO sub-vocabularies, i.e. cellular component, biological process, and molecular function, using STRING software (https://string-db.org) [97]. Codon usage workflow analysis, based on the GC content, GC2 (GC content at the second codon position), GC3s (GC content at the third site position), CBI (codon bias index), ENC (effective codon number), and RSCU (relative synonymous codon usage), was done by the DnaSP program [98]. Finally, clustering the data and drawing the heat maps were performed using Pearson correlation and the Mev4.0 software [99].

### Gene expression analysis based on RNA-seq data

The expression profiles of the *EgMTP* genes of six organs/tissues types (i.e. young and mature leaves, shoot tips, phloem, immature xylem, and xylem) from *E. grandis* TAG0014 clonal genotype were identified. Also, the expression profiles of MTPs tension wood xylem, upright control xylem (cambium), immature xylem, mature xylem, phloem, young leaves, mature leaves and shoot tips tissues from F1 Hybrid, *E. grandis* × *E. urophylla* (GUSAP1 clonal genotype), were identified using RNA-seq data obtained from EucGenIE (http://eucgenie.bi.up.ac.za) [100–103]. The expression data from biotic-treated *Eucalyptus* species were obtained from the EucGenIE database under the following conditions. The compatible third-generation *E. nitens* seedling stems were inoculated with *Phytophthora cinnamomi*, and mock inoculation was performed with sterile cV8 agar plugs over five-week post-inoculation from 1.5 cm above and below the inoculation sites [104]. Also, the two-year-old compatible and incompatible *E. grandis*, GC540, and TAG5 genotypes were grown in a field cage insectarium, respectively. After four months, the *E. grandis* clonal replicates were split into uninfected and infested *Leptocybe invasa* groups. This required the gall wasp and RNA of three biological replicates of six plants which were individually extracted from leaf midrib tissue harboring insect ovipositor sites after seven days [105]. In another experiment, the stems of one-year-old clones of the compatible (susceptible) clone *E. grandis* ZG14 and the incompatible (resistant) clone *E. grandis* TAG5 were inoculated with* Chrysoporthe austroafricana* (fungal pathogen) strain CMW2113 causing *Eucalyptus* stem canker [106]. Subsequently, the *MTP* gene expression was also analyzed as a unit of normalized transcripts per million (TPM) (fragments per kilo-base). The changes in the gene expression of *EgMTP* genes were analyzed using Pearson correlation and a complete linkage algorithm.

Developmental and stress-associated expression data were used to draw hierarchical clustering according to log_2_ values of different vegetative organs/tissues and were clustered into a heat map by MeV 4.0 software [99].

### Growth conditions and heavy metal treatments

In general, the seeds of *E. grandis* were obtained from the Seed and Plant Improvement Institute of Iran (SPII). First, the seeds were subjected to the disinfection process, submerged in 70% alcohol for 15 s, and then presoaked in benomyl 1 × 1000 solution for 20 min. Then, to eliminate any interaction related to soil and to control all conditions required for accessing any nutrients, the germination of seeds was carried out in 1–2 mm silica that was thoroughly washed and sterilized in an oven and transferred into individual pots containing PittMoss soil to continue their growth with optimum circumstances for 2 additional months. Different heavy metal concentrations of 50, 100, and 300 mM of CuSO_4_ and 10. 50 and 200 mM of Cd (NO_3_)_2_ were performed on the two-month-old seedlings treated in normal Hoagland's nutrient solution. The experiment was performed in a completely randomized design with three replications. The sampling of leaves and roots of plantlets was performed after 18 days of metal exposure. Then, they were harvested and directly preserved in liquid nitrogen and stocked at -80 °C until further analyses.

### RNA isolation, cDNA synthesis, and quantitative real-time PCR analysis (qRT-PCR)

pBiozol (Sigma) reagent was utilized for the total RNA isolation from all treated seedlings (roots and leaves), and subsequently, first-strand cDNA synthesis was performed using a Kiagene Kit (Fermentas GmbH, St. Leon-Rot, Germany). Primer3 software (http://frodo.wi.mit.edu/primer3/) was used to analyze the specific primers of the three candidate genes: *EgMTP5*, *EgMTP6*, *EgMTP1*, and *Elongation factor S-II* was used as a reference gene (Table 5). Quantitative real-time PCR (qRT-PCR) analysis was carried out in a total reaction mixture of 20 μL containing the following reagents: 10 μL of RealQ Plus 2 × Master Mix Green (containing SYBR Green Dye, Ampliqon, Denmark), 1.5 μL of cDNA, 0.25 μM of each primer, and 1.5 μL of PCR-grade water. qPCR reaction conditions were carried out as follows: 15 min at 95 °C, 15 s at 95 °C, 60 s at 60 °C for each primer in 45 cycles, and 15 s at 72 °C. The differential expression level was investigated by the 2^−ΔΔCT^ method with three technical and biological replications for each specimen [107].

**Table 5:** All sequences of primers used in the present study

Primer name	Primer sequences
EgMTP5-F	5-AGTTTGCCTGCACGTTCT-3
EgMTP5-R	3–5-TGC CAC TGA AAC TAG TCC CAA
EgMTP6-F	5-GTATTGCCTGGCATGCTCT-3
EgMTP6-R	5-GTGCTCCATGTCAACTCCA-3
EgMTP11-F	5-GCCATCATAGCCTCCACAT-3
EgMTP11-R	5-GGTTGCATCCGTTTCTTTCC-3
Elongation factor S-II-F	5- TCCAATCCGAGTCGCTGTCATTGT-3
Elongation factor S-II-R	5-TGATGAGCCTCTCTGGTTTGACCT-3

### Statistical analysis

Experimental results were the means of three replicates of heavy metal treatments. The data were analyzed by analysis of variance (ANOVA) to assess the significant differences between treatments using Duncan's multiple range tests (*p* ≤ 0.05). Also, the values were expressed as mean ± standard deviation (SD).

## Supplementary Information

**Additional file 1: Supplementary Table 1.**

## Data Availability

The datasets generated and/or analysed during the current study are available in TAIR (http://arabidopsis.org), RGAP (http://rice.plantbiology.msu.edu) and EucGenIE (https://eucgenie.org/) repository.

## Abbreviations

MTPs: Metal tolerance proteins
CDFs: Cation diffusion facilitators
PGDD: Plant genome duplication database
CREs: *cis*-Acting regulatory elements
GO: Gene ontology annotation
CUP: Codon usage pattern
qRT-PCR: Quantitative real-time PCR
TMDs: Transmembrane domains
