# Supplementary material for: Genome-wide identification and expression profile analysis of metal tolerance protein gene family in Eucalyptus grandis under metal stresses
Source: BMC Plant Biol. 2023 May 6;23:240. doi: 10.1186/s12870-023-04240-9 (PMC10163719; doi:10.1186/s12870-023-04240-9)
Supplement: Supplementary file 1 — Additional file 1: Supplementary Table 1. [file 12870_2023_4240_MOESM1_ESM.docx]

Supplementary Table 1: *Cis*‐regulatory element in the upstream of the three *EgMTP* sub-families. Mn-MTP (violet), Zn/Fe-MTP (blue) and Zn-MTP (green), are divided into four groups depending on their putative major cellular functions, i.e., light-responsive (yellow), regulation of plant development (blue), phytohormone-responsive (orange), environmental stress- responsive (green).

|  | *Eg*  *MTP8.1* | *Eg*  *MTP8.2* | *Eg*  *MTP9.3* | *Eg*  *MTP11.2* | *Eg*  *MTP11.1* | *Eg*  *MTP9.2* | *Eg*  *MTP9.1* | *Eg*  *MTP6* | *Eg*  *MTP7* | *Eg*  *MTP1.6* | *Eg MTP1.2* | *Eg*  *MTP1.4* | *Eg*  *MTP1.1* | *Eg*  *MTP1.3* | *Eg*  *MTP1.5* | *Eg*  *MTP1.7* | *Eg*  *MTP2* | *Eg*  *MTP12* | *Eg*  *MTP4* | *Eg*  *MTP5* |  |
| --- | --- | --- | --- | --- | --- | --- | --- | --- | --- | --- | --- | --- | --- | --- | --- | --- | --- | --- | --- | --- | --- |
| 3 |  | 1 |  |  |  |  |  |  |  |  | 1 |  |  |  |  |  |  |  |  | 1 | AuxR-core |
| 6 |  | 1 |  |  |  | 2 | 2 |  |  |  | 1 |  |  |  |  |  |  | 1 |  | 1 | P-box |
| 48 | 8 | 2 | 1 | 2 | 11 | 2 | 6 | 1 | 2 | 2 | 3 |  | 1 |  | 1 |  | 3 | 5 | 2 |  | ABRE |
| 34 |  | 2 | 2 |  | 4 | 5 | 6 |  | 1 | 1 | 1 | 3 |  | 1 | 3 | 2 | 1 | 1 |  | 1 | CGTCA-motif |
| 33 |  |  | 2 |  | 4 | 5 | 1 |  | 1 | 1 | 1 | 3 |  | 1 | 3 | 2 | 1 |  | 2 | 1 | TGACG-motif |
| 10 |  |  | 1 |  | 1 | 1 |  |  | 1 |  |  |  |  | 1 |  | 1 | 1 |  |  | 2 | TCA-element |
| 6 | 1 |  |  |  | 1 |  |  |  | 1 | 1 |  |  |  |  |  |  |  |  | 2 |  | GARE-motif |
| 3 |  |  | 1 |  |  |  |  |  |  |  |  |  |  | 1 |  |  |  |  | 1 |  | TATC-box |
| 11 |  |  |  |  |  | 3 |  |  | 1 |  |  | 1 |  | 1 | 3 |  | 1 |  | 1 |  | TGA-element |
| 12 | 1 | 1 | 1 |  | 1 | 2 | 1 |  | 1 | 1 | 1 |  |  |  |  |  |  | 1 |  | 2 | GATA-motif |
| 9 |  |  | 1 |  |  | 1 | 1 |  | 1 |  |  |  |  | 1 |  | 2 | 1 |  |  | 1 | TCCC-motif |
| 5 |  |  |  |  |  |  | 2 |  |  |  |  |  |  |  | 1 | 1 |  | 1 |  | 1 | Sp1 |
| 26 | 1 | 3 | 2 |  |  | 2 | 1 | 1 | 3 |  |  |  |  | 7 | 3 |  |  | 1 |  | 1 | Box 4 |
| 27 | 3 | 1 |  | 1 | 3 | 4 | 2 | 1 | 1 | 1 | 2 | 1 | 1 | 1 | 1 | 1 | 1 | 2 | 1 |  | G-Box |
| 29 | 5 |  | 2 | 2 | 8 |  |  | 2 |  | 1 | 2 |  | 1 |  |  |  | 1 | 2 | 1 |  | G-box |
| 1 |  |  |  |  |  |  |  |  |  |  |  |  |  |  |  |  | 1 |  |  |  | ATC-motif |
| 8 |  | 1 |  |  |  |  |  | 1 | 1 |  |  | 1 |  |  |  | 1 | 1 | 2 |  |  | GT1-motif |
| 1 |  |  |  |  |  |  |  |  |  |  |  |  |  |  |  |  | 1 |  |  |  | L-box |
| 10 |  | 1 | 1 |  |  |  |  | 1 | 1 | 1 | 1 | 1 | 1 |  |  | 1 |  |  |  | 1 | AE-box |
| 3 | 1 |  |  |  |  |  |  |  |  |  |  | 1 |  |  |  | 1 |  |  |  |  | ACE |
| 12 |  |  |  | 2 |  | 1 |  |  |  | 1 | 1 | 1 | 1 | 1 | 2 | 1 | 1 |  |  |  | TCT-motif |
| 7 |  |  |  | 1 | 1 | 1 |  |  |  |  |  |  |  |  | 1 |  |  | 3 |  |  | I-box |
| 5 |  | 1 |  |  | 1 |  |  |  | 1 |  |  |  |  |  | 1 |  |  | 1 |  |  | MRE |
| 2 |  |  |  |  |  |  |  | 1 |  |  |  |  |  | 1 |  |  |  |  |  |  | 3-AF1 binding site |
| 1 |  |  |  |  |  |  | 3 |  |  |  |  |  |  | 1 |  |  |  |  |  |  | ATCT-motif |
| 4 |  |  |  |  |  |  |  |  |  |  |  |  |  | 1 |  |  |  |  |  |  | GT1-motif |
| 1 |  |  |  |  |  |  |  |  |  |  |  | 1 |  |  |  |  |  |  |  |  | ACA-motif |
| 1 |  |  |  |  |  |  | 1 |  |  |  | 1 |  |  |  |  |  |  |  |  |  | chs-CMA2a |
| 2 |  |  |  |  |  |  |  |  |  |  |  |  |  |  |  |  |  | 1 |  |  | chs-CMA1a |
| 1 |  |  |  | 1 |  |  |  |  |  |  |  |  |  |  |  |  |  |  |  |  | GTGGC-motif |
| 1 |  |  |  | 1 |  |  |  |  |  |  |  |  |  |  |  |  |  |  |  |  | Gap-box |
| 4 |  |  |  |  |  |  | 1 |  |  | 1 | 1 | 1 |  |  |  |  |  |  |  | 1 | circadian |
| 30 |  | 1 | 1 |  | 1 |  |  |  | 1 | 5 | 4 | 2 | 2 | 2 |  | 3 | 2 |  | 2 | 3 | ARE |
| 1 |  |  |  |  |  |  |  |  |  |  |  |  |  |  |  |  | 1 |  |  |  | MBSI |
| 4 |  |  |  |  | 1 |  |  |  |  |  |  |  | 1 |  |  |  | 2 |  |  |  | TC-rich repeats |
| 6 | 1 |  | 1 |  | 2 |  | 1 |  | 1 |  |  |  |  |  |  |  |  | 1 |  |  | GC-motif |
| 3 | 1 |  |  |  |  |  |  |  |  |  |  |  |  |  | 1 |  |  |  |  |  | MBS |
| 21 | 2 |  | 4 |  | 1 | 1 | 1 |  |  | 3 | 3 |  |  | 1 |  | 3 | 2 |  |  | 1 | LTR |
| 15 |  |  | 2 | 1 | 1 | 1 |  | 1 |  | 3 | 2 | 1 |  |  |  |  |  | 1 |  | 1 | O2-site |
| 8 |  |  |  |  | 1 |  | 1 |  |  |  | 1 | 1 | 1 | 1 |  | 1 |  |  | 2 |  | MBS |
| 6 | 1 | 1 | 1 |  |  |  |  |  |  |  |  |  |  |  | 1 | 1 |  |  |  |  | CAT-box |
| 1 |  |  |  | 1 |  |  |  |  |  |  |  |  |  |  |  |  |  |  |  |  | HD-Zip 1 |
| 5 |  | 1 |  |  |  |  | 2 |  |  |  | 1 | 1 |  |  |  |  |  |  | 2 |  | GCN4_motif |
